# Rational Design, Synthesis, and Biological Evaluation of Novel Thiazole/Thiazolidinones Multitarget Anti-Human Immunodeficiency Virus Molecules

**DOI:** 10.3390/ph18030298

**Published:** 2025-02-21

**Authors:** Christophe Tratrat, Anthi Petrou, Maria Fesatidou, Micheline Haroun, Mohamad Chohan, Athina Geronikaki

**Affiliations:** 1 Department of Pharmaceutical Sciences, College of Clinical Pharmacy, King Faisal University, Al-Ahsa 31982, Saudi Arabia; mharoun@kfu.edu.sa; 2 Department of Pharmaceutical Chemistry, School of Pharmacy, Aristotle University of Thessaloniki, 54124 Thessaloniki, Greece; fesa.maria@gmail.com; 3 Department of Biomedical Sciences, College of Clinical Pharmacy, King Faisal University, Alhofuf 36362, Saudi Arabia; mshwhan@kfu.edu.sa

**Keywords:** anti-HIV molecules, multitarget drugs, HIV-1 RT, RNase H, Docking

## Abstract

**Background:** HIV-1 RT inhibitors were the first drugs approved to treat AIDS and remain key components of highly active antiretroviral therapy (HAART). While HAART effectively suppresses viral replication and slows disease progression, it has limitations, including long-term side effects and the emergence of drug-resistant strains, highlighting the need for new treatments. **Objectives:** Based on our previous experience, and insights from existing inhibitors of HIV-1 RT and RNase H, we aim to design and synthesize safer, multifunctional molecules. **Methods:** Using molecular docking studies, these compounds will incorporate pharmacophores targeting multiple stages of the HIV life cycle to enhance efficacy, reduce resistance, and improve pharmacokinetics. The compounds were synthesized via a one-pot three component reaction. The synthesized compounds were identified using spectroscopy and tested in vitro for activity against key HIV targets, including RNA-dependent DNA polymerase (RDDP) and RNAse H. **Results:** Among the synthesized compounds, several demonstrated strong inhibitory activity, with compound **11** showing IC_50_ values comparable to the reference drug Nevirapine, and compound **4** exhibiting dual inhibition of both RT and RNase H activities. **Conclusions:** These findings emphasize the importance of a multidisciplinary approach, combining computational modeling with experimental validation, to identify promising leads for therapeutic development.

## 1. Introduction

AIDS has been a global public health problem since its appearance. It is a disease caused by the infection and destruction of T-cells by the human immunodeficiency virus (HIV) resulting in the diminution of the immune system allowing life-threatening opportunistic infections [1].

Although the transmission of the virus is limited to sexual contact, blood transfusions, or from mother to newborn, due to the mass movement of the population, the virus has spread rapidly worldwide and nowadays AIDS is a very serious problem. With a treatment still lacking, along with the growing resistance of the available antiretroviral drugs, the development of new, more effective, safer drugs is an urgent need.

HIV is a retrovirus, which uses RNA instead of DNA to multiply. The process involves multi-steps and begins with the attachment of the virus to the host cell surface followed by the synthesis of the viral DNA by action of the reverse transcriptase (RT), then the integrase (IN) will incorporate the viral DNA into the host DNA. Transcription and translation of the hybrid DNA leads to the production of viral proteins and genome that gather together to form new viral particles able to infect other cells [2].

From the HIV life cycle, it is clear that the inhibition of RT and RNAse H can stop the virus infection at a very early stage. Thus, these enzymes are considered major drug targets; nowadays, RT is a common target for almost half of all approved anti-HIV drugs [3].

Reverse transcriptase (RT) is a multifunctional heterodimeric enzyme characterized by the subunits of 66 and 51 kDa (p66/p51) consisting of three distinct catalytic activities. The first one is the RNA-dependent DNA-polymerase (RDDP) activity where the pro-viral DNA is produced from the viral RNA. The second catalytic activity is the cleavage of the RNA portion of the RNA/DNA hybrid by the ribonuclease H (RNase H), and the last activity involved the DNA-dependent DNA-polymerase (DDDP) activity responsible for the DNA synthesis [4].

Therefore, DNA polymerase and RNase H activities are crucial steps in virus multiplication [5]. Both are found in two different domains of the p66 RT subunit where the RNase H domain and the DNA polymerase domain are located at the C-terminus and at the N-terminus, respectively, with a distance separation of 60 Å. Both RT catalytic sites are dependent to each other in which mutations in the RNase H domain affect the DNA polymerase activity, and vice versa [6]

Owing to their significant role in viral replication, both RDDP and RNase H represent major targets for the discovery of novel RT inhibitors that could also be effective against multi-drug resistant strains [7] and could also reduce the administered drug dose [8].

**Figure 1:**
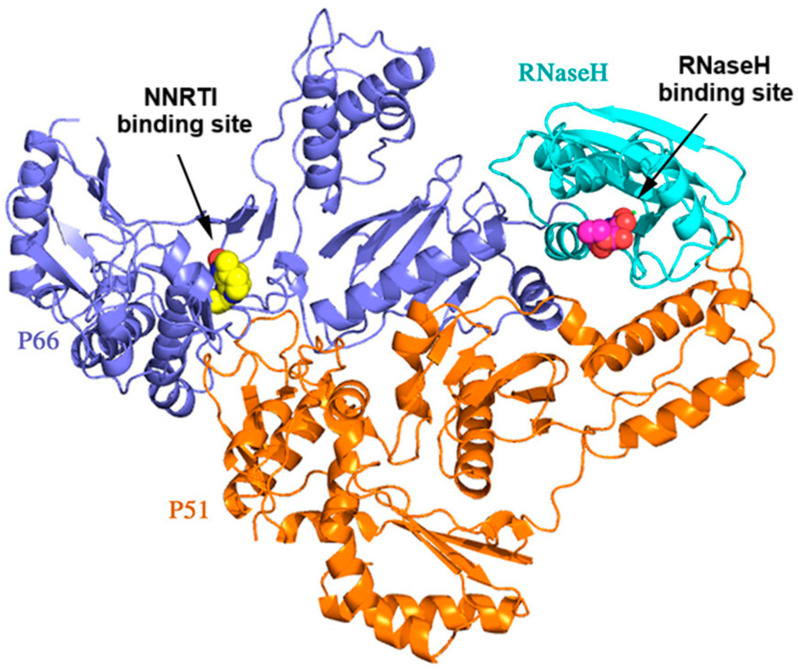
Overall view of HIV-1 reverse transcriptase and ligand binding sites of RNase H and NNRTIs.

There are two well-known classes of drugs targeting the DNA polymerase: the nucleoside/nucleotide RT inhibitors (NRTIs/NtRTIs) and the non-nucleoside RT inhibitors (NNRTIs) [8]. NRTIs/NtRTIs are prodrugs activated by viral thymidine kinase, which act as a DNA chain termination, whereas the NNRTIs are chemically diverse compounds which inhibit DNA polymerase in a non-competitive manner. NNRTIs have gained a significant place in clinical use due to their fewer side effects and lower cytotoxicity compared to NRTIs that bind to host polymerases [9]. Crystallographic studies have shown that the NNRTIs binding region is located in an allosteric center 10 Å far from the active center of the enzyme and contains four important residual amino acids, the hydrophobic Leu234, Val179, and Tyr181 and the hydrophilic Lys101 (Figure 1). NNRTIs bind to these amino acids in the form of a “butterfly” with two “wings” forming p-p interactions with the aromatic side chains of these amino acids. An important feature of classical inhibitors (efavirenz, nevirapine, etravirine, etc.) is the formation of a conventional hydrogen bonding interaction with the residue Lys101 [10].

**Figure 2:**
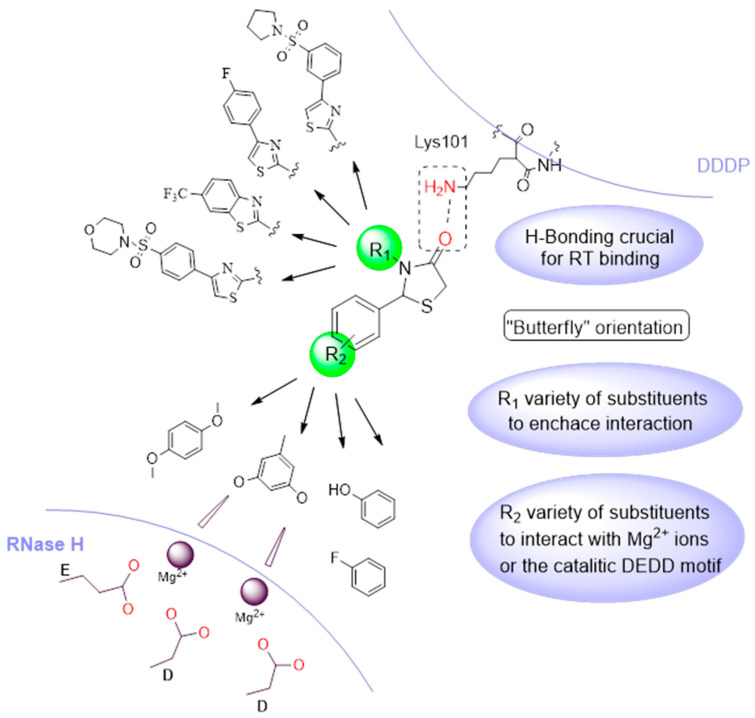
The design of novel RT/ RNase H dual inhibitor. The essential H-bond crucial to RT target (red) and the conformation of the molecule remains unchanged. The catalytic DEDD motif that coordinates the two Mg^2+^ ions (purple), a critical structural component for RNase H binding.

On the other hand, there are still no drugs in clinical use targeting the RNase H, thus representing a more challenging molecular target, although some molecules demonstrated inhibition action in preclinical testing, [11] including allosteric site and metal-chelating active site. The HIV RT RNase H active site, consisting of four conserved catalytic acidic residues (Asp443, Asp498, Asp549, and Glu478, DEDD), is located in a cavity where the amino acid residue H539 residue is found to be essential in enzyme activity [12]. Another catalytic domain, which is primordial for enzyme function, is the catalytic DEDD motif in which two Mg^2+^ ions are involved in binding interaction. Metal-chelating active site inhibitors interact with the two metal cations in the RNase H active site blocking the access of the metals, thereby preventing the metal-catalyzed hydrolysis of the phosphodiester bond in the RNA strand (Figure 2). Based on the literature [13,14] the minimum pharmacophore characteristics of these inhibitors are the presence of a β-diketo acid group capable of chelating the two Mg^2+^ ions and a benzyl group.

Based on our previous experience and considering the important role of NNRTIs in combination with antiretroviral therapy as well as the literature data on the activity of 2,3-diaryl-thiazolidin-4-one derivatives [15,16,17,18] in this study we attempt the develop novel compounds that will be effective multitarget inhibitors of HIV-1 RT and RNase H, more resistant to virus mutations, and important tools for the treatment of AIDS.

## 2. Results and Discussion

### 2.1. Rational Design of Compounds

An important tool in designing drug-like molecules is the use of molecular docking representing a cost-effective and time-saving process. A major prerequisite for a structure-based drug design approach is the molecular atomic knowledge of the drug targets through the availability of 3D X-ray protein structures, which represent the starting point for rational drug design by determining the topography of the binding site to be completed by the structure of the ligand.

In this research, we used modern approaches in medicinal chemistry combining structural biology and computational chemistry. Our aim was to design new compounds with the ability to target both sites of RT enzyme by taking into account the required pharmacophores. In particular, they were specifically designed on the basis of the following structural features and interactions provided to improve their drug action:

- The presence of a 1,3-thiazolidin-4-one moiety, due to its derivatives been reported as strong RT inhibitors [15,16,17,18]. Moreover, some derivatives have shown a inhibition mechanism different to that of most NNRTIs, which may enable them to act against resistant strains [15].
- Remarkably the binding site of NNRTI consists of mainly hydrophobic contacts while only a few hydrogen bonding interactions are needed. The presence of the conventional hydrogen bonding contact between the inhibitor (Figure 2) and the residue Lys101 significantly constitutes to a hydrophilic interaction [19]. This type of H-bond contact may positively contribute to the binding affinity of NNRTI and will serve as an anchor for the inhibitor adopting the most appropriate conformation with the aim of maximizing hydrophobic interaction contacts in the binding site. The O atom of the C=O group of the thiazolidinone moiety can play this role.
- The presence of a thiazole or benzothiazole moiety and aromatic rings facilitate hydrophobic interactions via π-π interactions with the residual amino acids side chains—Tyr181, Tyr188, Trp229, Phe227, Val106, Pro236, Leu100, and Leu234—enhancing the RT inhibition.
- The presence of a benzyl group at position 2 of the thiazolidinone moiety helps the molecule to obtain the “butterfly” configuration required to bind to the allosteric center of NNRTIs and inhibit RT.
- The presence of a dyad of –OH as a substituent of the phenyl group to be involved in the chelation process with two magnesium ions along with an existing hydrophobic phenyl group, which represents one of the most important pharmacophore needed for RNase H binding (Figure 2).
- The presence of a halogen substituent and mainly chlorine in benzothiazole, has been found to increase the inhibitory activity of the compounds [20]. Halogens form electrostatic interactions, which are stronger than H-bonds, and thereby a more stable complex is formed resulting in higher inhibition [21].

On the basis of the above information, we have designed several compounds subjected to molecular docking simulation along with the determination of biological activity spectrum and investigated their pharmacokinetic profile. Thus, fifteen compounds were selected for their preparation and the biological evaluation in vitro with the best probability of being potent multi-target inhibitors to both RDDP and RNase H.

### 2.2. Docking Studies

In order to select the most promising compounds for further research, we performed molecular docking simulations on a variety of designed compounds to identify the most interesting compounds with high binding affinity against both sites of the HIV-RT enzyme.

For the docking studies, the enzyme of HIV-1 reverse transcriptase in complex with TMC125 inhibitor (PDB code: 3MEC) and RT RNase H active site in complex with N-hydroxythienopyrimidine-2,4-dione inhibitor (PDB code: 6AOC), were used.

All compounds were subjected to the docking process as a racemic mixture. The best conformers were chosen on the basis of the RMSD values and the estimated stronger negative free binding energy. Out of the 85 designed compounds (SF, 1,3-5-), the top 15 exhibited the most favorable binding energies to both sites of the enzyme simultaneously, as shown in Table 1. The selection of these conformers was also guided by the number of intermolecular interactions observed within the enzyme’s active site. These include hydrogen bonds, aromatic interactions, and hydrophobic interactions, particularly with the Lys101 residue of HIV-1 reverse transcriptase. For the RNase H active site, the interactions involve Mg^2+^ ions or the conserved catalytic acidic residues (Asp443, Asp498, Asp549, and Glu478, comprising the DEDD motif), which play a critical role in enzyme activity. These top 15 compounds were then selected for further studies.

In all cases, as a first step, the primary docking was performed in order to validate the docking protocol. All ligands, as they were bonded in the crystal structures, were extracted and docked back into the analogous binding pocket in order to determine the ability of docking to replicate the position and orientation of each inhibitor in the crystal structure, in terms of root mean square deviation (RMSD) value.

### 2.3. Chemistry

**Scheme 1:**
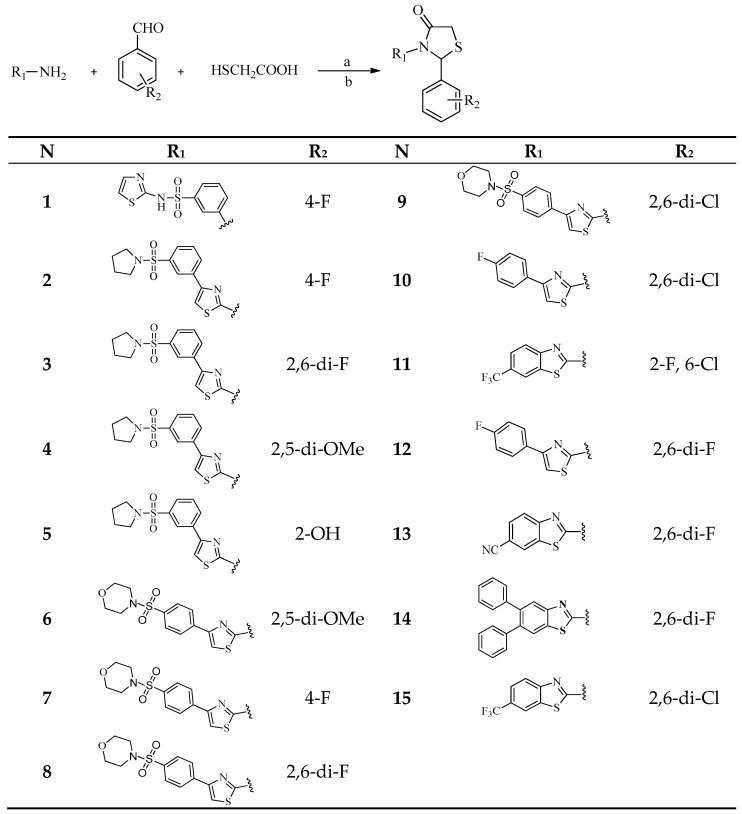
General synthetic procedure. Reagents and conditions: (a) conventional method: dry toluene, reflux for 18–32 h; (b) microwave-assisted technique: 100 °C, power 100 W, 30 min.

As detailed in our previous publications, the compounds were synthesized using a one-pot method, as shown in Scheme 1 [15,22]. For compounds **4**, **6**, **8**, and **14**, a microwave-assisted one-pot procedure was employed (Scheme 1).

All products were synthesized in a racemic mixture and were characterized by various spectroscopic methods (^1^H NMR and ^13^C NMR, Supplementary Materials).

In the ^1^H NMR spectra, signals at 8.12–7.00 ppm, 6.72–7.15 ppm, and 3.85–4.13 ppm were assigned to the aromatic protons, N-CH-S, and -CH_2_ protons, respectively. It is worth mentioning that the protons of the CH_2_ group of the thiazolidine ring exhibited two distinct peaks as a doublet. In the case of methoxy-substitution on the benzene ring, a singlet at 3.76–3.95 ppm was observed, and for the hydroxy-derivatives, a broad peak at 5.32–5.35 ppm was observed.

In the ^13^C NMR spectra, a peak attributed to the C=O group was observed at 170–171 ppm, while for C-2 of benzothiazole ring at 163–165 ppm and for C-2 and C-5 of thiazolidine one moiety at 60–63 ppm and at 31–34 ppm. Finally, peaks attributed to the carbon atom of the phenyl ring attached to the hydroxyl group and of the -CF_3_ substitution in the benzothiazole ring appeared at 156 ppm and 129 ppm, respectively.

### 2.4. Drug-Likeness Properties

The calculated ADMET properties of the most active compounds are presented in Table 2 and for the rest of them in Table S2. One of the most critical challenges for oral medications is their ability to cross the intestinal epithelial barrier, which controls the rate and extent of human absorption and, consequently, their bioavailability. The Caco-2 permeability assay allows orally administered drugs prediction on how well the compound will be absorbed; a value > 8 × 10^−6^ cm/s indicates high permeability. For the pkCSM predictive model, a value > 0.90 indicates high Caco2 permeability. With the exception of compounds **2**–**6,13** and the reference drug Etravirine, the rest have higher values than this threshold, indicating good permeability. The positive values of compounds **1**, **5**, **10**, **15**, and Etravirine show that they can be transported across the cell membrane by the ATP-binding cassette (ABC) transporter, a component of P-glycoprotein.

The Volume of Distribution (VDss) indicates the extent to which a drug is evenly distributed throughout the body. A VDss value below −0.15 (log VDss < −0.15) suggests low distribution, while a VDss value above 0.45 (log VDss > 0.45) indicates high distribution. In this context, compounds **1**, **7**, **8**, and **14** are considered to have a low VDss. The blood–brain barrier (BBB) permeability reflects a substance’s ability to enter the brain. A logBB value greater than 0.3 typically signifies BBB penetration. However, with the exception of compounds **10** and **15**, the logBB values for all compounds indicate low BBB permeability. The majority of the compounds exhibit low permeability to the Central Nervous System (CNS), with compounds having a logPS value less than −3 considered unable to penetrate the CNS. However, compounds **10**, **11**, **12**, **14**, **15** and Etravirine, with logPS values greater than −3, may potentially penetrate the CNS.

Metabolism prediction suggested that most of the compounds are both substrates and inhibitors of CYP2D6 and CYP3A4. Moreover, compounds **1–8, 13** as well as reference drug Etravirine are predicted to show hepatotoxicity, while the others were non-toxic.

### 2.5. Biological Evaluation

#### 2.5.1. In Vitro Evaluation of HIV-1 RT RDDP Inhibitory Activity

All compounds were evaluated against RT-HIV activity. The results are reported in Table 3. The study revealed that four derivatives demonstrated good to excellent activity with an IC_50_ in the range of 0.32–2.23 μΜ. However, the remaining compounds showed moderate to low activity. The order of the compounds’ potency is as follows: Nevirapine >**11** > **4** > **6** > **1**. The best activity was shown by compound **11** (IC_50_ = 0.32 μΜ) which had the same potency as the reference drug Nevirapine (IC_50_ = 0.31 μΜ). Good activity was also observed for compounds **4** and **6** with sub-micromolar inhibition but they were less active than the reference drug Nevirapine.

The structure–activity relationship revealed that the presence of 2,5-di-OMe (**4**, **6**) and 2-F, 6-Cl (**11**) R_2_ substituents in the benzene ring showed enhanced activity compared to other compounds. Thus, replacing these substituents with others in the benzene ring led to reduced activity. For R_1_ substituents, the presence of a benzothiazole moiety (**11**) is more favorable for anti-HIV activity, followed by a pyrrolidine and morpholine moiety (**4**, **6**) at position 4 of the benzenesulfonamide group compared to the thiazole moiety (**10** and **12**). Therefore, the results clearly indicate that the activity is influenced not only by the type and position of the substituent on the benzene ring but also by the nature of the heterocyclic ring attached to the sulfonamide group.

**Figure 3:**
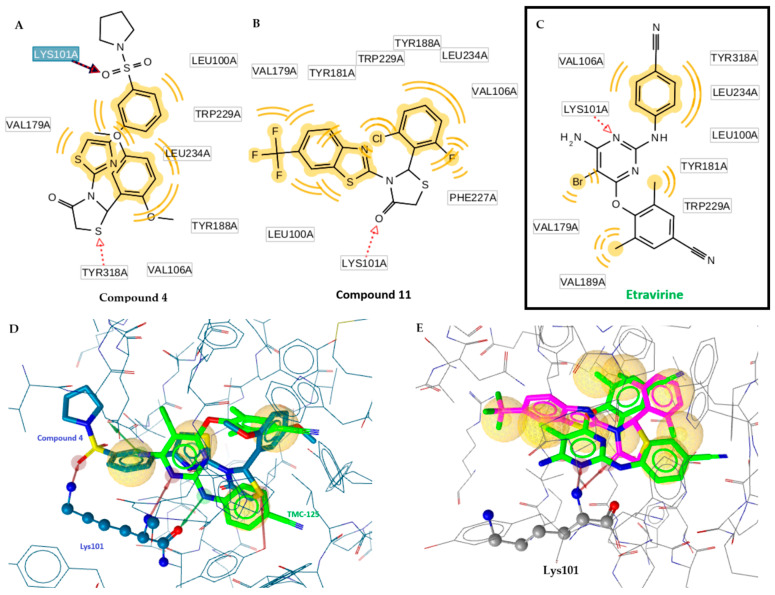
(**A**) A 2D interaction diagram of compound **4** docking pose. (**B**) A 2D interaction diagram of compound **11** docking pose. (**C**) A 2D interaction diagram of Etravirine inhibitor docking pose. (**D**) Superposition of compound **4** (blue) and Etravirine (green). (**E**) Superposition of compound **11** (magenta) and Etravirine (green) in active site of RT-HIV. Red dotted arrows indicate H-bonds and yellow spheres hydrophobic interactions.

Docking studies of highly active compounds **4** and **11** revealed their interactions with the enzyme’s active site, including hydrogen bond formation with the catalytic residue Lys101. Additionally, compound **4** established an extra hydrogen bond with Tyr318 and engages in hydrophobic interactions with residues Leu100, Trp229, Leu234, Tyr188, Val106, and Val179 (Figure 3). These interactions contribute to enhanced stabilization of the inhibitor–enzyme complex, correlating with the observed increase in inhibitory activity.

#### 2.5.2. RT RNase H Polymerase-Independent Cleavage Assay

Synthesized compounds were also evaluated for their inhibitory activity against RNase H enzyme and the results are presented in Table 4. Our results revealed that seven compounds out of fifteen exhibited interesting inhibition activity with IC_50_ values in the range of 1.47–25.37 μΜ. Among all compounds, the most active compound appeared to be compound **4** (IC_50_ 1.47 μΜ) followed by compound **6** (IC_50_ 2.50 μΜ).

On the basis of the structure–activity relationship, it is shown that the presence of 2,5-di-OMe substituent in the benzene ring is favorable for promoting inhibitory activity (**4** and **6**). Moreover, it seems that the pyrrolidine (**4**) and morpholine moieties (**6** and **9**) at position 4 of the benzenesulfonamide groups are beneficial. Thus, the results clearly indicate that the activity is influenced not only by the type of substituent and its location on the benzene ring but also by the type of heterocyclic ring attached to the sulfonamide group.

**Figure 4:**
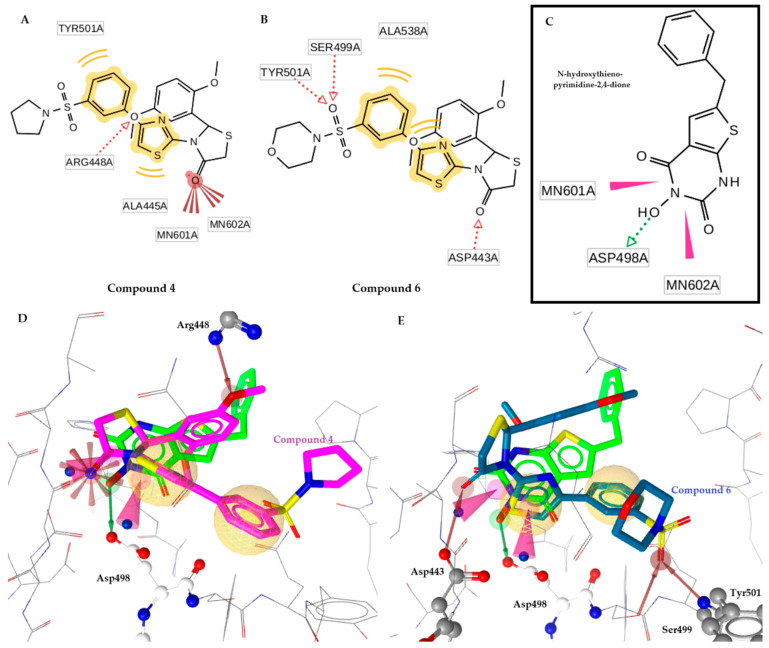
(**A**) A 2D interaction diagram of compound 4 docking pose. (**B**) A 2D interaction diagram of compound **6** docking pose. (**C**) A 2D interaction diagram of N-hydroxythieno-pyrimidine-2,4-dione inhibitor docking pose. (**D**) Superposition of compound **4** (pink) and N-hydroxythieno-pyrimidine-2,4-dione inhibitor (green) in active site of RT RNase H. (**E**) Superposition of compound **6** (blue) and N-hydroxythieno-pyrimidine-2,4-dione inhibitor (green) in active site of RT RNase HActive site. Hydrogen bonds are shown as red and green dotted arrows, hydrophobic interactions as yellow spheres, and Mg^2+^ ions as blue spheres.

Docking studies of the most active compound **4** interact with the enzyme’s active site, including hydrogen bond formation with Arg448 (O···H, 2.25 Å). Additionally, compound **4** interacts with Mg^2+^ (Figure 4). These interactions contribute to enhanced stabilization of the inhibitor–enzyme complex, correlating with the observed increase in inhibitory activity. Similarly, RDS1759, a well-characterized RNase H inhibitor, was shown to inhibit HIV-1 proliferation by targeting genomic RNA hydrolysis through interactions not only with Mg^2+^ but also with highly conserved residues within the RNase H domain [23].

While compound **6** exhibits a similar IC_50_ to compound **4**, Table 1 indicates that it does not engage in direct interactions with Mg^2+^ ions, suggesting alternative binding mechanisms. Notably, compound **6** forms three hydrogen bonds, including one with Asp443, a key residue in the catalytic motif of the enzyme, which plays a crucial role in stabilizing the binding pose and enhancing inhibitory potency. Additionally, compound **6** establishes more hydrophobic interactions compared to compound **4**, which may contribute to its binding affinity and overall activity, potentially compensating for the absence of Mg^2+^ coordination. Interestingly, ester derivative RDS1759 also selectively inhibits RNase H activity and viral replication in the low micromolar range, forming key interactions with residues Gln475, Asn474, and Tyr501 [23], similar to the hydrogen bonding and hydrophobic contributions observed for compound **6**. These findings reinforce the idea that RNase H inhibition is not solely driven by Mg^2+^ chelation but also by key interactions with conserved amino acids within the RNase H domain.

### 2.6. Assessment of Cellular Viability

**Figure 5:**
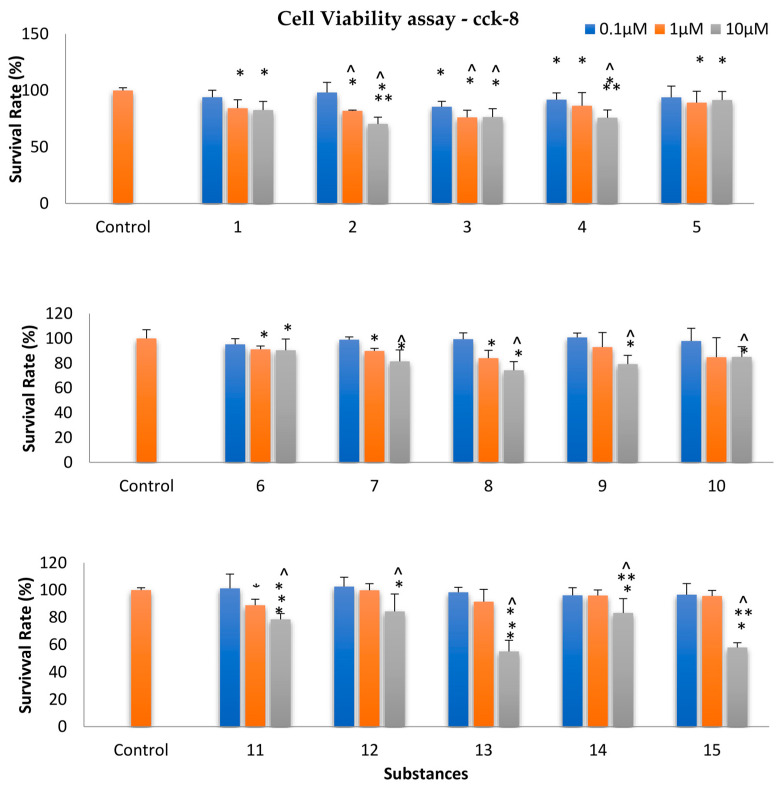
Survival rate (%) of MRC-5 cells exposed for 24 h under various concentrations of the substances. All values are presented as mean ± standard deviation (SD) of triplicate incubations. This with * represent the significant differences between each concentration and the control group. The symbol ^ represents the statistically significant difference between each concentration examined and the 0.1 µM for each compound. Similarly, the symbol ** represents the statistically significant difference in the higher concentration (10 μM) with the intermediate concentration (1 μM). The statistical tests used for this analysis were one way ANOVA and *t*-tests, with a significance level of *p* < 0.05.

For the evaluation of cytotoxicity, compounds **1**–**15** were tested in the human normal fetal lung fibroblast MRC-5 cell line using the CCK cell viability assay. Each substance was tested at three different concentrations: 0.1 μM, 1 μM, and 10 μM. It was observed that no notable negative impact was observed on these normal cells at the two lowest concentrations after an incubation period of 24 h (Figure 5). At the highest concentration of 10 µM, compounds **13** and **15** exhibited the highest cytotoxicity (with viabilities of 55.0% and 57.9%, respectively). However, since all viability percentages were above 50%, no CC_50_ value could be calculated. Hence, we can conclude that this series of compounds induces low cytotoxicity, allowing us to select the most promising compounds for antiviral evaluation.

## 3. Materials and Methods

### 3.1. Docking Studies

Molecular docking studies were performed using X-ray crystal structures of HIV-1 reverse transcriptase in complex with TMC125 inhibitor (PDB code: 3MEC) and RT RNase H active site in complex with N-hydroxythienopyrimidine-2,4-dione inhibitor (PDB code: 6AOC) were obtained from the protein data bank (PDB) [24] via AutoDock 4.2 program [25].

In particular, for the preparation of the ligand structures, all molecules were sketched in chemdraw 12.0 program. The geometry of the built compounds was optimized using the molecular mechanical force fields 94 (MMFF94) energy via program LigandScout (ver. 4.4.5) [26], partial charges were also calculated, conformers of each ligand were generated, and the one with the best conformation was maintained and saved as mol2 files and passed to ADT for pdbqt file preparation. A polar hydrogen was added to each structure, followed by computing Gasteiger and Kollman charges, and the torsions. The region of interest, used by Autodock4 for docking runs and by Autogrid4 for affinity grid maps preparation, was defined in such a way to comprise the whole catalytic binding site using a grid size of 50 × 50 × 50 xyz points with grid spacing of 0.375 Å.

For the docking simulation, default values of quaternation, translation, and torsion steps were applied. The Lamarckian Genetic Algorithm with default parameters was applied for minimization. The number of docking runs was 100. Upon completion of docking, the best poses were screened by examination of the binding energy (ΔGbinding, kcal/mol) and the number of clusters. In order to describe the ligand–binding pocket interactions, the top-ranked binding mode found by AutoDock in complex with the binding pocket of the enzyme was selected. The LigandScout (ver. 4.4.5) [26] was used for the graphical representations of all ligand-protein complexes.

Finally, before performing the docking studies, the co-crystallized ligands of the enzymes were removed and docked into the active site of the enzymes in order to validate the accuracy of the docking program. The results revealed that the docked ligands TMC125 and N-hydroxythienopyrimidine-2,4-dione inhibitor were superimposed on the co-crystallized bound ones with a root mean square deviation value (RMSD) of 0.90 Å and 1.17 Å, respectively, indicating the ratability of our docking protocol.

### 3.2. ADMET Properties

The pkCSM pharmacokinetics server, accessible at https://biosig.lab.uq.edu.au/pkcsm/prediction in Melbourne, Australia (accessed 13 October 2023, reanalysis 28 June 2025) [27], was employed to predict the ADMET properties, encompassing both physicochemical and pharmacological aspects of all designed compounds and the reference inhibitor N3. Initially, all molecules, including the reference drug Etravirine, were converted into SMILES (Simplified Molecular Input Line Entry Specification) format, which was then provided as input to the pkCSM pharmacokinetic server for prediction.

### 3.3. Chemistry

The melting points were determined using the MEL-TEMP II device (LAB Devices, Holliston, MA, USA). ^1^H NMR nuclear magnetic resonance spectra were acquired in DMSO-d_6_ and CDCl_3_ using an Agilent spectrometer operating at 500 MHz. Chemical shifts are reported in parts per million (ppm), with tetramethylsilane (TMS, δTMS = 0 ppm) serving as the internal standard. The reaction progress was monitored via thin layer chromatography, employing F254 silica gel chromatography plates (Merck, Darmstadt, Germany). All reagents and solvents were purchased from Aldrich Chemie (Steinheim, Germany) and were of high analytical purity. All compounds were purified to a high standard, and their purity was confirmed to be greater than 95%.

#### 3.3.1. General Procedure for the Synthesis of Thiazolidinones by a Conventional Method

The (hetero)aromatic amine (1.0 mmol) and either 2,6-dihalo-substituted benzaldehyde or monosubstituted benzaldehyde (1.2 mmol) were mixed and stirred in dry toluene under reflux conditions. Mercaptoacetic acid (2.0 mmol) was then added to the mixture. The reaction was further refluxed for 18–32 h, and then concentrated under reduced pressure until dry. The resulting residue was dissolved in ethyl acetate and sequentially washed with 5% aqueous citric acid, water, 5% aqueous sodium hydrogen carbonate, and brine. After drying over sodium sulfate, the organic layer was evaporated under reduced pressure to yield a crude product. This product was subsequently purified via column chromatography on silica gel, using a hexane–ethyl acetate mixture (8:2) as the eluent.

#### 3.3.2. Microwave Irradiation Experiments

For compounds **4**, **6**, **8**, and **14**, a microwave-assisted one-pot procedure was employed. All experiments involving microwave irradiation were conducted using a dedicated CEM-Discover monomode microwave apparatus, operating at a frequency of 2.45 GHz. The irradiation power ranged continuously from 0 to 300 W, with a standard absorbance level set at a maximum of 300 W. In a 10 mL reaction vial equipped with a stirring bar, the reagents, including aminobenzothiazole (1.5 mmol), an equimolar amount of 2,6-dihalosubstituted benzaldehyde (1.5 mmol), and mercaptoacetic acid (2.0 mmol) in absolute ethanol (3 mL) were combined. The vial was purged with argon for 30 s, tightly sealed with a Teflon septum, and then inserted into the microwave apparatus. Irradiation was carried out at a predetermined temperature ceiling of 80–100 °C, using 100 W as the maximum power, for a duration of 30 min. Subsequently, the reaction mixture was rapidly cooled to ambient temperature using gas jet cooling.

3-(2-(4-Fluorophenyl)-4-oxothiazolidin-3-yl)-N-(thiazol-2-yl)benzenesulfonamide (**1**), was previously reported [22].

2-(4-Fluorophenyl)-3-(4-(3-(pyrrolidin-1-ylsulfonyl)phenyl)thiazol-2-yl)thiazolidin-4-one (**2**).

Yield: 58%. Reaction time: 20 h. m.p. 207–209°C. R_f_: 0.62 (toluene/ethanol, 8/2). ^1^H NMR (500 MHz, DMSO-*d*_6_) δ 8.00–7.91 (m, 3H), 7.79 (d, *J* = 8.2 Hz, 2H), 7.58 (dd, *J* = 5.2, 8.5 Hz, 2H), 7.18 (t, *J* = 8.7 Hz, 2H), 6.79 (s, 1H), 4.37 (d, *J* = 16.5 Hz, 1H), 4.03 (d, *J* = 16.6 Hz, 1H), 3.12 (s, 4H), 1.61 (s, 4H). ^13^C NMR (126 MHz, DMSO-*d*_6_) δ 171.35 (C=O), 156.66, 147.30 138.17, 138.03, 135.49, 129.07, 129.01, 128.36 (2C), 126.65 (2C), 115.95, 115.77, 112.56, 109.98, 62.77, 48.27 (2C), 32.51, 25.14 (2C). Anal.Calcd. for C_22_H_20_FN_3_O_3_S_3_ (%): C, 53.97; H, 4.12; N, 8.58. Found (%): C, 53.92; H, 4.18; N, 8.91.

2-(2,6-Difluorophenyl)-3-(4-(3-(pyrrolidin-1-ylsulfonyl)phenyl)thiazol-2-yl)thiazolidin-4-one (**3**).

Yield: 58%. Reaction time: 22 h. m.p. 215–216°C. R_f_: 0.34 (toluene/ethanol, 8/2).^1^H NMR (500 MHz, DMSO-*d*_6_) δ 8.26 (s, 1H), 8.12 (s, 1H), 7.96–7.88 (m, 1H), 7.82–7.75 (m, 1H), 7.39 (dd, *J* = 4.6, 11.0 Hz, 1H), 7.16 (s, 3H), 7.00 (s, 1H, N-CH-S), 4.28–4.11 (d, *J* = 10.6 Hz, 1H,S-CH_2_), 3.55 (d, *J* = 10.6 Hz, 1H,S-CH_2_), 3.12 (s, 4H), 1.65–1.57 (m, 4H). ^13^C NMR (126 MHz, DMSO-*d*_6_) δ 171.19 (C=O), 163.21, 161.26, 156.18, 147.65, 135.11, 134.03, 133.01, 131.14, 131.00, 130.67, 129.20, 127.92, 127.86, 116.01, 115.84, 109.18, 61.19 (2C), 59.35, 34.29, 25.84 (2C). Anal.Calcd. for C_22_H_19_F_2_N_3_O_3_S_3_ (%): C, 52.06; H, 3.77; N, 8.28. Found (%): C, 52.03; H, 3.78; N, 8.31.

2-(2,5-Dimethoxyphenyl)-3-(4-(3-(pyrrolidin-1-ylsulfonyl)phenyl)thiazol-2-yl)thiazolidin-4-one (**4**).

Yield: 78%. Reaction time: 30 min (microwave-assisted one-pot procedure). m.p. 248–249 °C. R_f_: 0.64 (toluene/ethanol, 8/2).^1^H-NMR (500 MHz, DMSO-*d*_6_) δ 7.99–7.90 (m, 3H), 7.81–7.72 (m, 2H), 6.96 (d, *J* = 9.0 Hz, 2H), 6.86–6.80 (m, 1H), 6.79 (s, 1H), 4.18 (d, *J* = 16.3 Hz, 1H, S-CH_2_), 4.00 (d, *J* = 16.3 Hz, 1H, S-CH_2_), 3.33 (s, 6H, O-CH_3_), 3.18–3.04 (m, 4H), 1.64–1.53 (m, 4H). ^13^C NMR (126 MHz, DMSO-*d*_6_) δ 171.71 (C=O), 156.66 (C-OCH_3_), 153.25 (C-OCH_3_), 150.94, 147.23, 138.06 (C-SO_2_), 135.46, 129.75, 128.25 (2C), 126.62 (2C), 114.05, 113.16, 112.48 (2C), 56.74 (O-CH_3_), 55.79 (O-CH_3_), 49.03 (N-C-S), 48.25 (2C), 25.12 (3C). Anal.Calcd. for C_24_H_25_N_3_O_5_S_3_ (%): C, 54.22; H, 4.74; N, 7.90. Found (%): C, 54.17; H, 4.79; N, 7.81.

2-(2-Hydroxyphenyl)-3-(4-(3-(pyrrolidin-1-ylsulfonyl)phenyl)thiazol-2-yl)thiazolidin-4-one (**5**).

Yield: 65%. Reaction time: 32 h. m.p. 242–244 °C. R_f_: 0.68 (toluene/ethanol, 8/2). ^1^H NMR (500 MHz, DMSO-*d*_6_) δ 7.96–7.91 (m, 1H), 7.78–7.76 (m, 1H), 7.41 (dd, *J* = 1.7, 7.7 Hz, 2H), 7.04 (td, *J* = 1.8, 7.7 Hz, 2H), 6.79–6.67 (m, 4H), 5.57 (S, 1H, OH), 4.18 (d, *J* = 16.3 Hz, 1H, S-CH_2_), 4.00 (d, *J* = 16.3 Hz, 1H, S-CH_2_), 3.12 (d, *J* = 14.7 Hz, 4H), 2.92 (d, *J* = 14.6 Hz, 4H).^13^C NMR (126 MHz, DMSO-*d*_6_) δ 171.72 (C=O), 156.55 (C-OH), 153.20, 150.92, 147.20, 138.00, 135.44, 129.73 (2C), 128.25 (2C), 126.60 (2C), 114.02, 113.15, 112.29, 49.02 (N-C-S), 48.24 (2C), 25.14 (2C). Anal.Calcd. for C_22_H_21_N_3_O_4_S_3_ (%): C, 54.19; H, 4.34; N, 8.62. Found (%): C, 54.14; H, 4.30; N, 8.67.

2-(2,5-Dimethoxyphenyl)-3-(4-(4-(morpholinosulfonyl)phenyl)thiazol-2-yl)thiazolidin-4-one (**6**).

Yield: 85%. Reaction time: 30 min (microwave-assisted one-pot procedure). m.p. 225–226 °C. R_f_: 0.57 (toluene/ethanol, 8/2).^1^H NMR (500 MHz, DMSO-*d*_6_) δ 8.03–7.94 (m, 3H), 7.76–7.66 (m, 2H), 6.96 (d, *J* = 8.9 Hz, 1H), 6.84 (d, *J* = 1.2 Hz, 1H), 6.83–6.75 (m, 2H), 4.19 (d, 16.3 Hz, 1H, S-CH_2_), 4.01 (d, *J* = 16.3 Hz, 1H, S-CH_2_), 3.80 (s, 7H, CH_2_-O-CH_2_, O-CH_3_), 3.76–3.55 (m, 7H, CH_2_-N-CH_2_, O-CH_3_). ^13^C NMR (126 MHz, DMSO-*d*_6_) δ 171.74 (C=O), 156.71 (C-OCH_3_), 153.25(C-OCH_3_), 150.94, 147.13, 138.48 (C-SO_2_), 133.70, 129.73, 128.63 (3C), 126.69 (2C), 114.04, 113.16, 112.79, 65.69 (2C, CH_2_-O-CH_2_), 56.75 (S-CH-N), 55.79 (2C, O-CH_3_), 46.31 (2C, CH_2_-N-CH_2_), 33.12 (S-CH_2_). Anal.Calcd. for C_24_H_25_N_3_O_6_S_3_ (%):C, 52.63; H, 4.60; N, 7.67. Found (%): C, 52.61; H, 4.64; N, 7.65.

2-(4-Fluorophenyl)-3-(4-(4-(morpholinosulfonyl)phenyl)thiazol-2-yl)thiazolidin-4-one (**7**), was reported before [22].

2-(2,6-Difluorophenyl)-3-(4-(4-(morpholinosulfonyl)phenyl)thiazol-2-yl)thiazolidin-4-one (**8**).

Yield: 89%. Reaction time: 30 min (microwave-assisted one-pot procedure). m.p. 244–245 °C. R_f_: 0.60 (toluene/ethanol, 8/2). ^1^H NMR (500 MHz, DMSO-*d*_6_) δ 8.00 (s, 1H), 7.91–7.84 (m, 2H), 7.67 (dd, *J* = 6.6, 8.2 Hz, 3H), 7.41–7.28 (m, 2H), 4.29–4.11 (m, 2H, S-CH_2_), 3.60 (t, *J* = 4.6 Hz, 5H, CH_2_-O-CH_2_), 2.84 (t, *J* = 4.7 Hz, 4H,CH_2_-N-CH_2_). ^13^C NMR (126 MHz, DMSO-*d*_6_) δ 171.39 (C=O), 156.56 (2C, C-F), 146.87, 138.39 (C-SO_2_), 135.19, 133.88, 133.05, 131.16, 129.33, 128.64, 128.60, 126.51, 65.70 (2C, CH_2_-O-CH_2_), 59.36 (S-CH-N), 46.32 (2C, CH_2_-N-CH_2_), 34.27 (S-CH_2_). Anal.Calcd. for C_22_H_19_F_2_N_3_O_4_S_3_ (%): C, 50.47; H, 3.66; N, 8.03. Found (%): C, 50.42; H, 3.63; N, 8.11.

2-(2,6-Dichlorophenyl)-3-(4-(4-(morpholinosulfonyl)phenyl)thiazol-2-yl)thiazolidin-4-one (**9**), was reported before [22].

2-(2,6-Dichlorophenyl)-3-(4-(4-fluorophenyl)thiazol-2-yl)thiazolidin-4-one (**10**).

Yield: 58%. Reaction time: 19 h. m.p. 189–191 °C. R_f_: 0.60 (toluene/ethanol, 8/2).^1^H NMR (500 MHz, DMSO-*d*_6_) δ 7.72–7.62 (m, 3H), 7.60 (dd, *J* = 1.5, 7.8 Hz, 1H), 7.40–7.27 (m, 2H), 7.22–7.09 (m, 2H), 4.27–4.12 (m, 3H, CH_2_-C=O). ^13^C NMR (126 MHz, DMSO-*d*_6_) δ 171.19 (C=O), 156.19 (C-F), 147.65, 135.11, 134.03, 133.01, 131.14 (2C, C-Cl), 131.00, 129.20 (2C), 127.92, 127.86, 116.01, 115.84, 109.19, 59.35 (N-C-S), 34.28 (S-CH_2_). Anal.Calcd. for C_18_H_11_Cl_2_FN_2_OS_2_ (%): C, 50.83; H, 2.61; N, 6.59. Found (%): C, 50.79; H, 2.65; N, 6.70.

2-(2-Chloro-6-fluorophenyl)-3-(6-(trifluoromethyl)benzo[d]thiazol-2-yl)thiazolidin-4-one (**11**) was reported before [28].

Yield: 72%. Reaction time: 21 h. m.p. 202–204 °C. R_f_: 0.62 (toluene/ethanol, 8/2). ^1^H NMR (500 MHz, CDCl_3_) δ 8.07 (d, *J* = 1.6 Hz, 1H), 7.77 (d, *J* = 8.6 Hz, 1H), 7.62–7.57 (m, 1H), 7.19 (td, *J* = 5.6, 8.1 Hz, 2H), 7.08 (s, 1H), 6.90 (t, *J* = 9.9 Hz, 1H), 4.26 (d, *J* = 16.2 Hz, 1H), 4.03 (d, *J* = 16.2 Hz, 1H). ^13^C NMR (126 MHz, CDCl_3_) δ 171.61 (C=O), 158.03, 150.35, 135.77, 133.63, 132.76, 132.09 (2C), 129.76, 128.91, 128.69, 123.10 (2C, CF_3_), 122.08, 118.91, 59.77(N-CH-S), 34.69 (S-CH_2_). Anal.Calcd. for C_17_H_9_ClF_4_N_2_OS_2_ (%):C, 47.17; H, 2.10; N, 6.47. Found (%):C, 47.21; H, 2.08; N, 6.31.

2-(2,6-Difluorophenyl)-3-(4-(4-fluorophenyl)thiazol-2-yl)thiazolidin-4-one (**12**).

Yield: 68%. Reaction time: 18 h. m.p. 201–202 °C. R_f_: 0.64 (toluene/ethanol, 8/2). ^1^H NMR (500 MHz, DMSO-*d*_6_) δ 7.74–7.57 (m, 4H), 7.42–7.25 (m, 3H), 7.21–7.09 (m, 2H), 4.28–4.13 (m, 2H, CH_2_-C=O). ^13^C NMR (126 MHz, DMSO-*d*_6_) δ 171.19 (C=O), 156.19 (C-F), 147.65, 135.11, 134.03, 133.01, 131.14 (3C), 131.00, 129.20 (2C), 127.92 (2C), 127.87, 116.03, 115.85, 109.20, 59.35 (N-C-S), 34.28 (S-CH_2_). Anal.Calcd. for C_18_H_11_F_3_N_2_OS_2_ (%):C, 55.09; H, 2.83; N, 7.14. Found (%):C, 55.11; H, 2.84; N, 7.18.

2-(2-(2,6-Difluorophenyl)-4-oxothiazolidin-3-yl)benzo[d]thiazole-6-carbonitrile (**13**) was reported in [29].

2-(2,6-Difluorophenyl)-3-(5,6-diphenylbenzo[d]thiazol-2-yl)thiazolidin-4-one (**14**).

Yield: 72%. Reaction time: 30 m (microwave-assisted one-pot procedure). m.p. 205–206 °C. R_f_: 0.67 (toluene/ethanol, 8/2). ^1^H NMR (500 MHz, CDCl_3_) δ 7.48–7.58 (m, 4H), 7.50–7.37 (m, 6H), 7.38–7.28 (m, 3H), 6.93–6.84 (m, 2H), 6.73 (s, 1H), 4.03 (d, *J* = 18.3 Hz, 1H), 3.82 (d, *J* = 18.3 Hz, 1H). ^13^C NMR (126 MHz, CDCl_3_) δ 172.81, 162.32, 162.25, 160.31, 152.46, 149.75, 138.16, 135.82, 134.95, 133.53, 130.07, 129.94, 128.34 (2C), 126.21, 123.23, 116.96, 115.97 (3C), 112.35 (4C), 56.00, 55.97, 55.93, 33.92. Anal.Calcd. for C_28_H_18_F_2_N_2_OS_2_ (%): C, 67.18; H, 3.62; N, 5.60. Found (%): C, 67.20; H, 3.60; N, 5.58.

2-(2,6-Dichlorophenyl)-3-(6-(trifluoromethyl)benzo[d]thiazol-2-yl)thiazolidin-4-one (**15**).

Yield: 56%. Reaction time: 25 h. m.p. 189–191 °C. R_f_: 0.63 (toluene/ethanol, 8/2). ^1^H NMR (500 MHz, DMSO-*d*_6_) 8.32 (d, J = 1.6 Hz, 1H), 7.73 (d, J = 7.5 Hz, 1H), 7.60 (s, 1H), 7.38–7.27 (m, 3H), 6.78 (s, 1H), 4.03 (d, J = 18.7 Hz, 1H, CH_2_-C=O), 3.88 (d, J = 18.9 Hz, 1H, CH_2_-C=O). ^13^C NMR (126 MHz, DMSO-*d*_6_) δ 172.81, 154.72, 150.04, 136.08, 133.95, 127.90, 127.72, 126.83, 125.76, 124.83, 124.58, 123.62, 119.46, 119.43, 119.42 (3C), 55.90, 33.92. Anal.Calcd. for C_17_H_9_Cl_2_F_3_N_2_OS_2_ (%): C, 45.44; H, 2.02; N, 6.23. Found (%): C, 45.48; H, 2.00; N, 6.25.

### 3.4. Biological Evaluation

#### 3.4.1. In Vitro Evaluation of HIV-1 RT RDDP Inhibitory Activity

As described in our previous work [15], HIV-1 reverse transcriptase (RT) activity was measured using the Roche colorimetric immunoassay kit. DNA synthesis by RT was quantified after 2 h incubation at 37 °C. The reaction included PolyA:oligodT RNA template, recombinant HIV-1 RT, and various substrates, with biotin-DIG-labeled DNA detected via streptavidin-coated ELISA wells. Inhibitor activity was tested by pre-incubating RT with compounds before substrate addition. The absorbance at 405 nm was measured to calculate enzyme activity and IC_50_ values. Nevirapine was used as a reference drug. The experiment was performed in triplicate. The data analysis for obtaining the IC_50_ values and standard deviation (SD)was performed using nonlinear regression analysis, specifically fitting the dose–response curves to a sigmoidal model (log(inhibitor) vs. response) using the software GraphPad Prism 10.

#### 3.4.2. RT RNase H Polymerase-Independent Cleavage Assay

The HIV-1 RT-associated RNase H activity was measured following the protocol cited [30] by the detection and quantification of RNase H activity of reverse transcriptase (RT) enzyme with a hybrid substrate formed by a fluorescein labeled RNA annealed with Dabcyl DNA. In this assay the reaction mixture containing tris–HCl, pH 7.8, MgCl_2_, dithiothreitol (DTT), KCl, hybrid RNA/DNA (5′-GTTTTCTTTTCCCCCCTGAC-3′-fluorescein,5′-CAAAAGAAAAGGGGGGACUG-3′-Dabcyl), and RT were incubated for 1 h at 37 °C, the reaction was stopped by the addition of EDTA, and products were measured with a Victor 3 (Perkin PerkinElmer VICTOR X3 Multilabel Counter Microplate Reader, Cridersville, OH, USA ) at 490/528 nm. The experiment was performed in triplicate. IC_50_ values were determined through nonlinear regression analysis, where the dose–response curves were fitted to a sigmoidal model (log(inhibitor) vs. response) using GraphPad Prism 10 software.

### 3.5. Evaluation of Cellular Viability

The normal human fetal lung fibroblast MRC-5 cell line (SI , 7)was cultured under standard conditions. Compounds were tested at 10 μM, 1 μM, and 0.1 μM. Results are expressed as mean ± standard deviation (SD) from triplicate experiments [31].

## 4. Conclusions

This study underscores the potential of integrating traditional medicinal chemistry with computational techniques to develop novel HIV-1 reverse transcriptase (RT) inhibitors. By leveraging molecular docking studies and structure–activity relationships, we designed multifunctional compounds targeting both the RNA-dependent DNA polymerase (RDDP) and RNase H active sites. Among the synthesized compounds, several demonstrated strong inhibitory activity, with compound **11** showing IC_50_ values comparable to the reference drug Nevirapine, and compound **4** exhibiting dual inhibition of both RT and RNase H activities. These results highlight the critical role of specific structural features, such as 2,5-di-OMe substituents and heterocyclic moieties like benzothiazole, in enhancing anti-HIV activity.

The docking studies revealed key intermolecular interactions, including hydrogen bonding and hydrophobic stabilization within the enzyme’s active site, further validating the observed biological activity. Importantly, cytotoxicity evaluations demonstrated that the compounds exhibited low toxicity at relevant concentrations, suggesting their suitability for further optimization and in vivo studies. The findings emphasize the importance of a multidisciplinary approach, combining computational modeling with experimental validation, to identify promising leads for therapeutic development.

Future work will focus on refining these compounds to improve their pharmacokinetic properties and exploring their efficacy in combination with existing antiretroviral drugs.

## Figures and Tables

**Table 1:** Results of molecular docking studies of compounds with crystal structures of HIV-RT enzyme and RNase H active site.

No	HIV-1 Reverse Transcriptase (PDB ID:3MEC)		RT RNase H Active Site (PDB: 6AOC)	
	Binding Free Energy (kcal/mol)	Residues Involved in Hydrogen Bonds	Binding Free Energy (kcal/mol)	Residues Involved in Hydrogen Bonds and Interactions with Mg
1	−9.25	Lys101 (O···H, 3.25Å)	−8.85	Asp498 (O···H, 3.74Å)
2	−9.56	Lys101 (O···H, 3.15Å), Lys102 (O···H, 3.57Å),	−7.76	Asp443 (H···O, 3.25Å)
3	−9.43	Lys101 (O···H, 3.55Å), Lys102 (O···H, 3.71Å),	−7.81	Asp498 (O···H, 2.84Å)
4	−9.82	Lys101 (O···H, 3.55Å), Tyr318 (S···H, 2.31Å)	−9.53	Arg448 (O···H, 2.25Å) Mg^2+^-O
5	−8.03	Lys101 (O···H, 3.47Å)	−9.10	Mg^2+^-O
6	−8.57	Lys101 (O···H, 3.59Å)	−9.36	Tyr501(O···H, 3.52Å), Asp443 (O···H, 3.12Å), Ser449(O···H, 3.72Å)
7	−9.45	Lys101 (O···H, 2.40Å), Lys103 (O···H, 2.31Å)	−8.62	Asp498 (O···H, 2.90Å)
8	−9.31	Lys101 (O···H, 2.74Å)	−7.73	Tyr501(O···H, 2.75Å)
9	−8.50	Lys101 (O···H, 2.77Å), His235 (O···H, 2.89Å)	−9.03	Tyr501(O···H, 3.49Å), Asp443 (O···H, 3.71Å)
10	−8.81	Lys101 (halogen bond), Thr181 (halogen bond)	−8.46	Asp498 (O···H, 3.56Å)
11	−9.13	Lys101 (O···H, 2.54Å)	−7.70	Asn265 (O···H, 3.57Å)
12	−8.58	Lys101 (O···H, 3.76Å)	−8.65	Tyr501(O···H, 3.30Å), Asp443 (O···H, 3.89Å)
13	−9.52	Lys101 (O···H, 2.33Å)	−8.59	Asn265 (Ν···H, 3.52Å)
14	−7.93	Lys101 (O···H, 2.82Å)	−7.94	Asn265 (S···H, 3.15Å)
15	−8.51	Lys101 (O···H, 3.54Å)	−8.19	Gly444 (O···H, 3.64Å)
TMC-125	−8.24	Lys101 (N···H, 3.22Å)	-	-
N-hydroxythieno- pyrimidine-2,4-dione	-	-	−10.79	Asp498 (N···H, 2.54Å) Mg^2+^-N

**Table 2:** ADMET values for Etravirine and tested compounds.

Property	Model Name	Predicted Value				Unit
		Etravirine	4	6	11	
**Absorption**	Water solubility	−3.718	−5.545	−5.281	−6.527	Numeric (log mol/L)
**Absorption**	Caco2 permeability	0.694	0.73	0.588	1.085	Numeric (log Papp in 10^−6^ cm/s)
**Absorption**	Intestinal absorption (human)	86.939	99.051	100	90.963	Numeric (% Absorbed)
**Absorption**	Skin permeability	−2.737	−2.838	−2.827	−2.704	Numeric (log Kp)
**Absorption**	P-Glycoprotein substrate	Yes	No	No	No	Categorical (Yes/No)
**Absorption**	P-Glycoprotein I inhibitor	Yes	Yes	Yes	Yes	Categorical (Yes/No)
**Absorption**	P-Glycoprotein II inhibitor	Yes	Yes	Yes	Yes	Categorical (Yes/No)
**Distribution**	VDss (human)	0.051	0.127	−0.09	0.172	Numeric (log L/kg)
**Distribution**	Fraction unbound (human)	0	0.046	0.062	0.038	Numeric (Fu)
**Distribution**	BBB permeability	−0.13	−1.545	−1.739	0.246	Numeric (log BB)
**Distribution**	CNS permeability	−1.981	−2.936	−3.117	−1.451	Numeric (log PS)
**Metabolism**	CYP2D6 substrate	No	No	No	No	Categorical (Yes/No)
**Metabolism**	CYP3A4 substrate	Yes	Yes	Yes	Yes	Categorical (Yes/No)
**Metabolism**	CYP2D6 inhibitor	No	No	No	No	Categorical (Yes/No)
**Metabolism**	CYP3A4 inhibitor	Yes	Yes	Yes	Yes	Categorical (Yes/No)
**Metabolism**	CYP1A2 inhibitor	Yes	No	No	Yes	Categorical (Yes/No)
**Metabolism**	CYP2C19 inhibitor	Yes	Yes	Yes	Yes	Categorical (Yes/No)
**Metabolism**	CYP2C9 inhibitor	Yes	Yes	Yes	Yes	Categorical (Yes/No)
**Excretion**	Total Clearance	−0.425	0.467	0.819	0.135	Numeric (log ml/min/kg)
**Excretion**	Renal OCT2 substrate	No	Yes	Yes	Yes	Categorical (Yes/No)
**Toxicity**	AMES toxicity	No	No	No	No	Categorical (Yes/No)
**Toxicity**	Max. Tolerated dose (human)	0.467	−0.211	−0.205	−0.075	Numeric (log mg/kg/day)
**Toxicity**	hERG I inhibitor	No	No	No	No	Categorical (Yes/No)
**Toxicity**	hERG II inhibitor	Yes	Yes	Yes	Yes	Categorical (Yes/No)
**Toxicity**	Oral Rat Acute Toxicity (LD50)	2.699	2.871	1.528	2.985	Numeric (mol/kg)
**Toxicity**	Hepatotoxicity	Yes	Yes	Yes	No	Categorical (Yes/No)
**Toxicity**	Skin Sensitization	No	No	No	No	Categorical (Yes/No)

The different colors reffer to different properties of ADMET.

**Table 3:** % HIV-Inhibitory activity at 4 μΜ and IC_50_ values ± standard deviation (SD) of tested compounds.

Entry	Inhibition at 4 μM, (%)	IC_50_ (μM)	Entry	Inhibition at 4 μM, (%)	IC_50_ (μM)
**1**	68.30	2.23 ± 0.15	**9**	2.48	>4
**2**	8.36	>4	**10**	1.59	>4
**3**	24.27	>4	**11**	92.37	0.32 ± 0.03
**4**	86.59	0.58 ± 0.07	**12**	5.86	>4
**5**	45.20	>4	**13**	21.37	>4
**6**	81.28	0.72 ± 0.06	**14**	7.90	>4
**7**	11.98	>4	**15**	8.52	>4
**8**	32.96	>4	**Nevirapine**	93.84	0.31 ± 0.06

**Table 4:** Inhibition of RT RNase H polymerase-independent enzyme (IC_50_values ± standard deviation (SD)).

Entry	IC_50_ (μM)	Entry	IC_50_ (μM)
**1**	14.30 ± 1.3	**9**	9.01 ± 0.1
**2**	>50	**10**	>50
**3**	>50	**11**	25.37 ± 0.4
**4**	1.47 ± 0.2	**12**	>50
**5**	18.01 ± 2.1	**13**	16.40 ± 1.7
**6**	2.50 ± 0.3	**14**	>50
**7**	>50	**15**	>50
**8**	>50	**RDS1759 [23]**	7.3 ± 0.1

## Data Availability

Data are contained within the article and Supplementary Materials.

## Supplementary Materials

The following supporting information can be downloaded at: https://www.mdpi.com/article/10.3390/ph18030298/s1, Table S1. Smiles of compounds. Table S2. ADMET values for Etravirine and tested compounds. Table S3. Results of molecular docking studies of designed compounds with crystal structures of HIV-RT enzyme and RNase H active site. References [32,33] are cited in Supplementary Materials.
