# Supplementary material for: Rational Design, Synthesis, and Biological Evaluation of Novel Thiazole/Thiazolidinones Multitarget Anti-Human Immunodeficiency Virus Molecules
Source: Pharmaceuticals (Basel). 2025 Feb 21;18(3):298. doi: 10.3390/ph18030298 (PMC11946194; doi:10.3390/ph18030298)
Supplement: Supplementary file 1 [file pharmaceuticals-18-00298-s001.zip › pharmaceuticals-3444697-supplementary.pdf]

Article

Rational Design, Synthesis and Biological Evaluation of Novel Thiazole/Thiazolidinones Multitarget Anti-HIV Molecules

Christophe Tratrat [1,†], Anthi Petrou [2,†*], Maria Fesatidou[2], Micheline Haroun[1], Mohamad Chohan [3], Athina Geronikaki[2,*]

[1] Department of Pharmaceutical Sciences, College of Clinical Pharmacy, King Faisal University, Al-Ahsa 31982, Saudi Arabia; ctratrat@kfu.edu.sa (Ch. T.); mharoun@kfu.edu.sa (M. H).
[2] Department of Pharmaceutical Chemistry, School of Pharmacy, Aristotle University of Thessaloniki, 54124 Thessaloniki, Greece
[3] Department of biomedical sciences , College of clinical pharmacy, Saudi Arabia

* Correspondence: Department of Pharmaceutical Chemistry, School of Pharmacy, Aristotle University of Thessaloniki, 54124 Thessaloniki, Greece; anthi.petrou.thessaloniki1"gmail.com (A.P.); geronik@pharm.auth.gr (A.G.);
† These authors contributed equally to this work.

SUPPORTING INFORMATION

Table of contents:

1. SMILES of the 85 designed compounds
2. Drug-likeness properties
3. Results of molecular docking studies of designed compounds
4. 2D interaction diagrams of compounds **1–15** and reference compounds with the HIV-1 RT active site
5. 2D interaction diagrams of compounds **1–15** and reference compounds with the RNAase active site
6. ^{1}H NMR and ^{13}C NMR of compounds
7. MRC-5 cell line information

1. SMILES of the 85 designed compounds

Table S1. Smiles of compounds.

No	Smiles	No	Smiles
**1**	O=C1N([C@H](C2=C(Cl)C=CC=C2F)SC1)C3=NC4=C(S3)C(Cl)=CC=C4	**44 (7)**	O=S(C1=CC=CC(C2=CSC(N3[C@H](C4=CC=C(F)C=C4)SCC3=O)=N2)=C1)(N5CCOCC5)=O
**2**	O=C1N([C@H](C2=CC=C(OC)C=C2)SC1)C3=NC4=C(S3)C=C(F)C=C4	**45**	O=S(C1=CC=CC(C2=CSC(N3[C@H](C4=CC=C(O)C=C4)SCC3=O)=N2)=C1)(N5CCOCC5)=O
**3**	O=C1N([C@H](C2=CC=C(O)C=C2)SC1)C3=NC4=C(S3)C=C(F)C=C4	**46**	O=S(C1=CC=CC(C2=CSC(N3[C@H](C4=CC=C(Br)C=C4)SCC3=O)=N2)=C1)(N5CCOCC5)=O
**4**	O=C1N([C@H](C2=CC=C(Br)C=C2)SC1)C3=NC4=C(S3)C=C(F)C=C4	**47**	O=S(C1=CC=CC(C2=CSC(N3[C@H](C4=CC=C(Cl)C=C4)SCC3=O)=N2)=C1)(N5CCOCC5)=O
**5**	O=C1N([C@H](C2=C(Cl)C=CC=C2Cl)SC1)C3=NC4=C(S3)C=C(F)C=C4	**48**	O=S(C1=CC=CC(C2=CSC(N3[C@H](C4=C(O)C=CC=C4)SCC3=O)=N2)=C1)(N5CCOCC5)=O
**6**	O=C1N([C@H](C2=CC=C(Cl)C=C2Cl)SC1)C3=NC4=C(S3)C=C(Cl)C=C4	**49 (6)**	O=S(C1=CC=CC(C2=CSC(N3[C@H](C4=C(OC)C=CC(OC)=C4)SCC3=O)=N2)=C1)(N5CCOCC5)=O
**7**	O=C1N([C@H](C2=CC=C(Cl)C=C2)SC1)C3=NC4=C(S3)C=CC=C4Cl	**50**	O=S(C1=CC=CC(C2=CSC(N3[C@H](C4=CC(OC)=C(O)C(OC)=C4)SCC3=O)=N2)=C1)(N5CCOCC5)=O
**8**	O=C1N([C@H](C2=CC=C(O)C=C2)SC1)C3=NC4=C(S3)C=CC=C4Cl	**51 (8)**	O=S(C1=CC=CC(C2=CSC(N3[C@H](C4=C(F)C=CC=C4F)SCC3=O)=N2)=C1)(N5CCOCC5)=O
**9**	O=C1N([C@H](C2=CC=C(O)C=C2)SC1)C3=NC4=C(S3)C=CC=C4OC	**52**	O=S(C1=CC=CC(C2=CSC(N3[C@H](C4=C(Cl)C=CC=C4F)SCC3=O)=N2)=C1)(N5CCOCC5)=O
**10**	O=C1N([C@H](C2=CC=C(Cl)C=C2Cl)SC1)C3=NC4=C(S3)C=C(OC(F)(F)F)C=C4	**53 (9)**	O=S(C1=CC=CC(C2=CSC(N3[C@H](C4=C(Cl)C=CC=C4Cl)SCC3=O)=N2)=C1)(N5CCOCC5)=O
**11**	O=C1N([C@H](C2=C(Cl)C=CC=C2Cl)SC1)C3=NC4=C(S3)C=C(OC(F)(F)F)C=C4	**54**	O=S(C1=CC=CC(C2=CSC(N3[C@H](C4=C(Cl)C=C(Cl)C=C4)SCC3=O)=N2)=C1)(N5CCOCC5)=O
**12**	O=C1N(C2=NC3=CC=C(OC(F)(F)F)C=C3S2)[C@@H](SC1)C4=C(Cl)C=CC=C4F	**55**	O=S(C1=CC=CC(C2=CSC(N3[C@H](C4=C(Cl)C(Cl)=CC=C4)SCC3=O)=N2)=C1)(N5CCOCC5)=O
**13**	O=C1N([C@H](C2=CC=C([N+]([O-])=O)C=C2)SC1)C3=NC4=C(S3)C=C(OC(F)(F)F)C=C4	**56**	FC1=CC=C(C2=CSC(N3[C@H](C4=CC=CC=C4)SCC3=O)=N2)C=C1
**14**	O=S(NC1=NC=CS1)(C2=CC=CC(N3[C@H](C4=CC=CC=C4)SCC3=O)=C2)=O	**57**	FC1=CC=C(C2=CSC(N3[C@H](C4=CC=C(F)C=C4)SCC3=O)=N2)C=C1
**15 (1)**	O=S(NC1=NC=CS1)(C2=CC=CC(N3[C@H](C4=CC=C(F)C=C4)SCC3=O)=C2)=O	**58**	FC1=CC=C(C2=CSC(N3[C@H](C4=CC=C(O)C=C4)SCC3=O)=N2)C=C1
**16**	O=S(NC1=NC=CS1)(C2=CC=CC(N3[C@H](C4=CC=C(Cl)C=C4)SCC3=O)=C2)=O	**59**	FC1=CC=C(C2=CSC(N3[C@H](C4=CC=C(Cl)C=C4)SCC3=O)=N2)C=C1
**17**	O=S(NC1=NC=CS1)(C2=CC=CC(N3[C@H](C4=C	**60**	FC1=CC=C(C2=CSC(N3[C@H](C4=CC=C(Br)

	C=C(O)C=C4)SCC3=O)=C2)=O		C=C4)SCC3=O)=N2)C=C1
**18**	O=S(NC1=NC=CS1)(C2=CC=CC(N3[C@H](C4=CC=C(Br)C=C4)SCC3=O)=C2)=O	**61**	FC1=CC=C(C2=CSC(N3[C@H](C4=C(O)C=CC=C4)SCC3=O)=N2)C=C1
**19**	O=S(NC1=NC=CS1)(C2=CC=CC(N3[C@H](C4=C(OC)C=CC(OC)=C4)SCC3=O)=C2)=O	**62**	FC1=CC=C(C2=CSC(N3[C@H](C4=C(OC)C=CC(OC)=C4)SCC3=O)=N2)C=C1
**20**	O=C1N([C@H](C2=C(F)C=CC=C2F)SC1)C3=NC4=C(S3)C=C(C(F)(F)F)C=C4	**63 (12)**	FC1=CC=C(C2=CSC(N3[C@H](C4=C(F)C=CC=C4F)SCC3=O)=N2)C=C1
**21 (11)**	O=C1N([C@H](C2=C(Cl)C=CC=C2F)SC1)C3=NC4=C(S3)C=C(C(F)(F)F)C=C4	**64**	FC1=CC=C(C2=CSC(N3[C@H](C4=C(Cl)C=CC=C4F)SCC3=O)=N2)C=C1
**22 (15)**	O=C1N([C@H](C2=C(Cl)C=CC=C2Cl)SC1)C3=NC4=C(S3)C=C(C(F)(F)F)C=C4	**65 (10)**	FC1=CC=C(C2=CSC(N3[C@H](C4=C(Cl)C=CC=C4Cl)SCC3=O)=N2)C=C1
**23**	O=C1N([C@H](C2=C(OC)C=CC(OC)=C2)SC1)C3=NC4=C(S3)C=C(C(F)(F)F)C=C4	**66**	FC1=CC=C(C2=CSC(N3[C@H](C4=C(Cl)C(Cl)=CC=C4)SCC3=O)=N2)C=C1
**24**	O=C1N([C@H](C2=CC=C(O)C=C2)SC1)C3=NC4=C(S3)C=C(C(F)(F)F)C=C4	**67**	FC1=CC=C(C2=CSC(N3[C@H](C4=C(Cl)C=C(Cl)C=C4)SCC3=O)=N2)C=C1
**25**	O=C1N([C@H](C2=CC=C(F)C=C2)SC1)C3=NC4=C(S3)C=C(C(F)(F)F)C=C4	**68**	FC1=CC=C(C2=CSC(N3[C@H](C4=CC(OC)=C(O)C(OC)=C4)SCC3=O)=N2)C=C1
**26**	O=C1N([C@H](C2=CC=C(Br)C=C2)SC1)C3=NC4=C(S3)C=C(C(F)(F)F)C=C4	**69**	FC1=CC=C(C2=CSC(N3[C@H](C4=CC(OC)=C(O)C=C4)SCC3=O)=N2)C=C1
**27**	O=C1N([C@H](C2=CC(OC)=C(O)C(OC)=C2)SC1)C3=NC4=C(S3)C=C(C(F)(F)F)C=C4	**70**	FC1=CC=C(C2=CSC(N3[C@H](C4=CC(OC)=C(I)C(OC)=C4)SCC3=O)=N2)C=C1
**28**	O=C1N([C@H](C2=C(OC)C=CC(OC)=C2)SC1)C3=NC4=C(S3)C=C(C#N)C=C4	**71**	O=C1N([C@H](C2=CC=C(F)C=C2)SC1)C3=NC4=C(S3)C=C(C5=CC=CC=C5)C(C6=CC=CC=C6)=C4
**29**	O=C1N([C@H](C2=CC=C(O)C=C2)SC1)C3=NC4=C(S3)C=C(C#N)C=C4	**72**	O=C1N([C@H](C2=CC=C(Cl)C=C2)SC1)C3=NC4=C(S3)C=C(C5=CC=CC=C5)C(C6=CC=CC=C6)=C4
**30 (13)**	O=C1N(C2=NC3=CC=C(C#N)C=C3S2)[C@@H](SC1)C4=C(F)C=CC=C4F	**73**	O=C1N([C@H](C2=CC=C(O)C=C2)SC1)C3=NC4=C(S3)C=C(C5=CC=CC=C5)C(C6=CC=CC=C6)=C4
**31**	O=C1N([C@H](C2=CC=C(F)C=C2)SC1)C3=NC4=C(S3)C=C(C#N)C=C4	**74**	O=C1N([C@H](C2=CC=C(Br)C=C2)SC1)C3=NC4=C(S3)C=C(C5=CC=CC=C5)C(C6=CC=CC=C6)=C4
**32**	O=C1N([C@H](C2=CC=C(Br)C=C2)SC1)C3=NC4=C(S3)C=C(C#N)C=C4	**75**	O=C1N([C@H](C2=CC(OC)=C(O)C(OC)=C2)SC1)C3=NC4=C(S3)C=C(C5=CC=CC=C5)C(C6=CC=CC=C6)=C4
**33**	O=S(C1=CC=CC(C2=CSC(N3[C@H](C4=CC=CC=C4)SCC3=O)=N2)=C1)(N5CCCC5)=O	**76**	O=C1N([C@H](C2=CC(OC)=C(O)C=C2)SC1)C3=NC4=C(S3)C=C(C5=CC=CC=C5)C(C6=CC=CC=C6)=C4
**34 (2)**	O=S(C1=CC=CC(C2=CSC(N3[C@H](C4=CC=C(F)C=C4)SCC3=O)=N2)=C1)(N5CCCC5)=O	**77**	O=C1N([C@H](C2=CC(OC)=C(I)C(OC)=C2)SC1)C3=NC4=C(S3)C=C(C5=CC=CC=C5)C(C6=CC=CC=C6)=C4
**35**	O=S(C1=CC=CC(C2=CSC(N3[C@H](C4=CC=C(Br)C=C4)SCC3=O)=N2)=C1)(N5CCCC5)=O	**78**	O=C1N([C@H](C2=C(OC)C=CC(OC)=C2)SC1)C3=NC4=C(S3)C=C(C5=CC=CC=C5)C(C6=CC=CC=C6)=C4
**36**	O=S(C1=CC=CC(C2=CSC(N3[C@H](C4=CC=C(O)C=C4)SCC3=O)=N2)=C1)(N5CCCC5)=O	**79 (14)**	O=C1N([C@H](C2=C(F)C=CC=C2F)SC1)C3=NC4=C(S3)C=C(C5=CC=CC=C5)C(C6=CC=CC=C6)=C4
**37 (5)**	O=S(C1=CC=CC(C2=CSC(N3[C@H](C4=C(O)C=CC=C4)SCC3=O)=N2)=C1)(N5CCCC5)=O	**80**	O=C1N([C@H](C2=C(Cl)C=CC=C2F)SC1)C3=NC4=C(S3)C=C(C5=CC=CC=C5)C(C6=CC=C

			C=C6)=C4
**38 (4)**	O=S(C1=CC=CC(C2=CSC(N3[C@H](C4=C(OC)C=CC(OC)=C4)SCC3=O)=N2)=C1)(N5CCCC5)=O	**81**	O=C1N([C@H](C2=C(Cl)C=CC=C2Cl)SC1)C3=NC4=C(S3)C=C(C5=CC=CC=C5)C(C6=CC=CC=C6)=C4
**39 (3)**	O=S(C1=CC=CC(C2=CSC(N3[C@H](C4=C(F)C=CC=C4F)SCC3=O)=N2)=C1)(N5CCCC5)=O	**82**	O=C1N([C@H](C2=C(Cl)C(Cl)=CC=C2)SC1)C3=NC4=C(S3)C=C(C5=CC=CC=C5)C(C6=CC=CC=C6)=C4
**40**	O=S(C1=CC=CC(C2=CSC(N3[C@H](C4=C(Cl)C=CC=C4F)SCC3=O)=N2)=C1)(N5CCCC5)=O	**83**	O=C1N([C@H](C2=C(Cl)C=C(Cl)C=C2)SC1)C3=NC4=C(S3)C=C(C5=CC=CC=C5)C(C6=CC=CC=C6)=C4
**41**	O=S(C1=CC=CC(C2=CSC(N3[C@H](C4=C(Cl)C=CC=C4Cl)SCC3=O)=N2)=C1)(N5CCCC5)=O	**84**	O=C1N([C@H](C2=C(Cl)C=CC=C2)SC1)C3=NC4=C(S3)C=C(C5=CC=CC=C5)C(C6=CC=CC=C6)=C4
**42**	O=S(C1=CC=CC(C2=CSC(N3[C@H](C4=CC(OC)=C(O)C(OC)=C4)SCC3=O)=N2)=C1)(N5CCCC5)=O	**85**	O=C1N([C@H](C2=CC(Cl)=CC=C2)SC1)C3=NC4=C(S3)C=C(C5=CC=CC=C5)C(C6=CC=CC=C6)=C4
**43**	O=S(C1=CC=CC(C2=CSC(N3[C@H](C4=CC=CC=C4)SCC3=O)=N2)=C1)(N5CCOCC5)=O		

2. Drug-Likeness Properties

The calculated ADMET properties of the most active compounds are presented in table 2 and for the rest of them in table S2. One of the most critical challenges for oral medications is their ability to cross the intestinal epithelial barrier, which controls the rate and extent of human absorption and, consequently, their bioavailability. The Caco-2 permeability assay is used to predict how well orally administered drugs will be absorbed; a value > $8x10^{-6}$ cm/s indicates high permeability. For pkCSM predictive model a value > 0.90 indicates high Caco2 permeability. With the exception of compounds **2-6, 13** and the reference drug Etravirine, the rest of them have higher values than this threshold, indicating good permeability. The positive values of compounds **1**, **5, 10, 15** and Etravirine showsthat they can be transported across the cell membrane by the ATP-binding cassette (ABC) transporter, a component of P-glycoprotein.

The Volume of Distribution (VDss) indicates the extent to which a drug is evenly distributed throughout the body. A VDss value below -0.15 (log VDss< -0.15) suggests low distribution, while a VDss value above 0.45 (log VDss> 0.45) indicates high distribution. In this context, compounds **1, 7, 8, 14** are considered to have a low VDss. Blood-brain barrier (BBB) permeability reflects a substance's ability to enter the brain. A logBB value greater than 0.3 typically signifies BBB penetration. However, with the exception of compounds **10** and **15**, the logBB values for all compounds indicate low BBB permeability. The majority of compounds exhibit low permeability to the Central Nervous System (CNS), with compounds having a logPS value less than -3 considered unable to penetrate the CNS. However, compounds **10, 11, 12, 14, 15** and etravirine, with logPS values greater than -3, may potentially penetrate the CNS.

Metabolism prediction suggested that most of compounds are both substrates and inhibitors of CYP2D6 and CYP3A4. Moreover, compounds **1-8, 13** as well as reference drug Etravirine are predicted to show hepatotoxicity, while the others were non-toxic.

Table S2. ADMET values for Etravirine and tested compounds.

Property	Model name	Predicted value								Unit
		Etravirine	1	2	3	4	5	6	7	
Absorption	Water solubility	-3.718	-4.863	-5.414	-5.587	-5.545	-4.953	-5.281	-5.13	Numeric (log mol/L)
Absorption	Caco2 permeability	0.694	0.901	0.591	0.668	0.73	0.702	0.588	1.014	Numeric (log Papp in 10^{-6}cm/s)
Absorption	Intestinal absorption (human)	86.939	92.371	95.963	95.673	99.051	94.484	100	97.147	Numeric (% Absorbed)
Absorption	Skin permeability	-2.737	-2.876	-2.766	-2.799	-2.838	-2.899	-2.827	-2.787	Numeric (log Kp)
Absorption	P-Glycoprotein substrate	Yes	Yes	No	No	No	Yes	No	No	Categorical (Yes/No)
Absorption	P-Glycoprotein I inhibitor	Yes	Yes	Yes	Yes	Yes	Yes	Yes	Yes	Categorical (Yes/No)
Absorption	P-Glycoprotein II inhibitor	Yes	Yes	Yes	Yes	Yes	Yes	Yes	Yes	Categorical (Yes/No)
Distribution	VDss (human)	0.051	-0.262	-0.016	-0.064	0.127	0.153	-0.09	-0.241	Numeric (log L/kg)
Distribution	Fraction unbound (human)	0	0.109	0.097	0.056	0.046	0.091	0.062	0.11	Numeric (Fu)

Distribution	BBB permeability	-0.13	-1.023	-1.302	-1.503	-1.545	-1.211	-1.739	-1.497	Numeric (log BB)
Distribution	CNS permeability	-1.981	-2.281	-2.082	-2.796	-2.936	-2.204	-3.117	-2.897	Numeric (log PS)
Metabolism	CYP2D6 substrate	No	No	No	No	No	No	No	No	Categorical (Yes/No)
Metabolism	CYP3A4 substrate	Yes	Yes	Yes	Yes	Yes	Yes	Yes	Yes	Categorical (Yes/No)
Metabolism	CYP2D6 inhibitor	No	No	No	No	No	No	No	No	Categorical (Yes/No)
Metabolism	CYP3A4 inhibitor	Yes	Yes	Yes	Yes	Yes	Yes	Yes	Yes	Categorical (Yes/No)
Metabolism	CYP1A2 inhibitor	Yes	Yes	No	No	No	No	No	No	Categorical (Yes/No)
Metabolism	CYP2C19 inhibitor	Yes	Yes	Yes	Yes	Yes	Yes	Yes	Yes	Categorical (Yes/No)
Metabolism	CYP2C9 inhibitor	Yes	Yes	Yes	Yes	Yes	Yes	Yes	Yes	Categorical (Yes/No)
Excretion	Total Clearance	-0.425	-0.135	0.131	0.231	0.467	0.281	0.819	0.484	Numeric (log ml/min/kg)
Excretion	Renal OCT2 substrate	No	No	Yes	Yes	Yes	No	Yes	Yes	Categorical (Yes/No)
Toxicity	AMES toxicity	No	No	No	No	No	No	No	No	Categorical (Yes/No)
Toxicity	Max. Tolerated dose (human)	0.467	0.116	-0.474	-0.172	-0.211	-0.198	-0.205	-0.482	Numeric (log mg/kg/day)
Toxicity	hERG I inhibitor	No	No	No	No	No	No	No	No	Categorical (Yes/No)
Toxicity	hERG II inhibitor	Yes	Yes	Yes	Yes	Yes	Yes	Yes	Yes	Categorical (Yes/No)
Toxicity	Oral Rat Acute Toxicity (LD50)	2.699	2.73	2.507	2.838	2.871	2.807	1.528	2.461	Numeric (mol/kg)
Toxicity	Hepatotoxicity	Yes	Yes	Yes	Yes	Yes	Yes	Yes	Yes	Categorical (Yes/No)
Toxicity	Skin Sensitization	No	No	No	No	No	No	No	No	Categorical (Yes/No)

**Property**	**Model name**	**Predicted value**								**Unit**
		**8**	**9**	**10**	**11**	**12**	**13**	**14**	**15**	
Absorption	Water solubility	-5.296	-5.667	-6.327	-6.527	-5.711	-4.773	-4.903	-6.978	Numeric (log mol/L)
Absorption	Caco2 permeability	1.25	0.59	1.042	1.085	1.1	0.881	1.022	1.095	Numeric (log Papp in 10^{-6}cm/s)
Absorption	Intestinal absorption (human)	96.857	94.976	92.183	90.963	94.064	100	96.416	90.083	Numeric (% Absorbed)
Absorption	Skin permeability	-2.801	-2.778	-2.607	-2.704	-2.67	-2.864	-2.735	-2.673	Numeric (log Kp)

Absorption	P-Glycoprotein substrate	No	No	Yes	No	No	No	No	Yes	Categorical (Yes/No)
Absorption	P-Glycoprotein I inhibitor	Yes	Yes	Yes	Yes	Yes	Yes	Yes	Yes	Categorical (Yes/No)
Absorption	P-Glycoprotein II inhibitor	Yes	Yes	Yes	Yes	Yes	Yes	Yes	Yes	Categorical (Yes/No)
Distribution	VDss (human)	-0.274	-0.086	0.158	0.172	-0.092	-0.069	-0.923	0.352	Numeric (log L/kg)
Distribution	Fraction unbound (human)	0.067	0.041	0.036	0.038	0.087	0.087	0.357	0.031	Numeric (Fu)
Distribution	BBB permeability	-1.698	-1.632	0.64	0.246	0.308	-0.991	0.133	0.372	Numeric (log BB)
Distribution	CNS permeability	-2.977	-2.006	-1.398	-1.451	-1.434	-2.404	-1	-1.421	Numeric (log PS)
Metabolism	CYP2D6 substrate	No	No	No	No	No	No	No	No	Categorical (Yes/No)
Metabolism	CYP3A4 substrate	Yes	Yes	Yes	Yes	Yes	Yes	Yes	Yes	Categorical (Yes/No)
Metabolism	CYP2D6 inhibitor	No	No	No	No	No	No	No	No	Categorical (Yes/No)
Metabolism	CYP3A4 inhibitor	Yes	Yes	No	Yes	Yes	Yes	No	Yes	Categorical (Yes/No)
Metabolism	CYP1A2 inhibitor	No	No	Yes	Yes	Yes	No	No	Yes	Categorical (Yes/No)
Metabolism	CYP2C19 inhibitor	Yes	Yes	Yes	Yes	Yes	Yes	Yes	Yes	Categorical (Yes/No)
Metabolism	CYP2C9 inhibitor	Yes	Yes	Yes	Yes	Yes	Yes	No	No	Categorical (Yes/No)
Excretion	Total Clearance	0.584	0.538	0.01	0.135	0.055	0.452	0.113	0.158	Numeric (log ml/min/kg)
Excretion	Renal OCT2 substrate	Yes	Yes	Yes	Yes	Yes	No	No	Yes	Categorical (Yes/No)
Toxicity	AMES toxicity	No	No	No	No	No	No	No	No	Categorical (Yes/No)
Toxicity	Max. Tolerated dose (human)	-0.176	-0.469	-0.062	-0.075	0.216	0.424	0.549	-0.113	Numeric (log mg/kg/day)
Toxicity	hERG I inhibitor	No	No	No	No	No	No	No	No	Categorical (Yes/No)
Toxicity	hERG II inhibitor	Yes	Yes	Yes	Yes	No	Yes	Yes	Yes	Categorical (Yes/No)
Toxicity	Oral Rat Acute Toxicity (LD50)	2.773	2.396	2.292	2.985	2.613	2.573	2.695	2.978	Numeric (mol/kg)
Toxicity	Hepatotoxicity	Yes	No	No	No	No	Yes	No	No	Categorical (Yes/No)
Toxicity	Skin Sensitization	No	No	No	No	No	No	No	No	Categorical (Yes/No)

3. **Table S3.** Results of molecular docking studies of designed compounds with crystal structures of HIV-RT enzyme and RNase H active site.

No	Binding free energy (kcal/mol)		No	Binding free energy (kcal/mol)	
	HIV-1 Reverse transcriptase (PDB ID:3MEC)	RT RNase H active site (PDB: 6AOC)		HIV-1 Reverse transcriptase (PDB ID:3MEC)	RT RNase H active site (PDB: 6AOC)
**1**	-6.28	-6.15	**44 (7)**	-9.45	-8.62
**2**	-6.23	-6.84	**45**	-6.20	-7.38
**3**	-7.02	-5.71	**46**	-5.75	-5.37
**4**	-5.19	-3.28	**47**	-5.62	-8.87
**5**	-7.42	-5.30	**48**	-4.98	-6.10
**6**	-7.53	-6.93	**49 (6)**	-8.57	-9.36
**7**	-8.55	-4.31	**50**	-7.70	-6.82
**8**	-5.29	-7.90	**51 (8)**	-9.31	-7.73
**9**	-6.53	-8.64	**52**	-8.84	-6.55
**10**	-6.38	-5.87	**53 (9)**	-8.50	-9.03
**11**	-9.06	-6.47	**54**	-8.26	-6.51
**12**	-7.36	-5.42	**55**	-7.69	-4.16
**13**	-5.93	-2.84	**56**	-6.33	-5.50
**14**	-5.45	-7.15	**57**	-6.91	-5.63
**15 (1)**	-9.25	-8.85	**58**	-6.90	-7.68
**16**	-7.21	-6.39	**59**	-8.85	-6.13
**17**	-6.39	-7.72	**60**	-5.72	-5.84
**18**	-5.69	-5.03	**61**	-7.15	-7.42
**19**	-6.36	-8.63	**62**	-5.18	-8.56
**20**	-6.33	-4.85	**63 (12)**	-8.58	-8.65
**21 (11)**	-9.13	-7.70	**64**	-7.93	-6.20
**22 (15)**	-8.51	-8.19	**65 (10)**	-8.81	-8.46
**23**	-7.66	-6.25	**66**	-6.83	-7.56
**24**	-6.63	-7.45	**67**	-6.59	-5.11
**25**	-6.60	-4.19	**68**	-5.17	-8.24
**26**	-6.20	-6.37	**69**	-6.45	-7.08
**27**	-6.72	-8.73	**70**	-6.45	-6.82
**28**	-6.39	-7.81	**71**	-6.40	-5.31
**29**	-6.40	-7.36	**72**	-6.86	-6.50
**30 (13)**	-9.52	-8.59	**73**	-5.34	-7.26
**31**	-7.18	-5.23	**74**	-5.43	-5.84
**32**	-5.29	-4.17	**75**	-5.39	-8.46
**33**	-7.80	-6.21	**76**	-6.97	-7.43
**34 (2)**	-9.56	-7.76	**77**	-5.20	-8.67
**35**	-5.98	-5.73	**78**	-6.05	-7.32
**36**	-6.39	-7.61	**79 (14)**	-7.93	-7.94
**37 (5)**	-8.03	-9.10	**80**	-7.16	-5.39
**38 (4)**	-9.82	-9.53	**81**	-6.30	-7.45
**39 (3)**	-9.43	-7.81	**82**	-7.53	-4.67

**40**	-7.69	-6.82	**83**	-6.37	-7.46
**41**	-7.35	-4.79	**84**	-5.38	-9.01
**42**	-6.31	-8.85	**85**	-6.79	-5.37
**43**	-6.70	-4.97			
**TMC-125**	-8.24		**N-hydroxythieno-pyrimidine-2,4-dione**		-10.79

4. **2D interaction diagrams of compounds 1–15 and reference compounds with the HIV-1 RT active site**

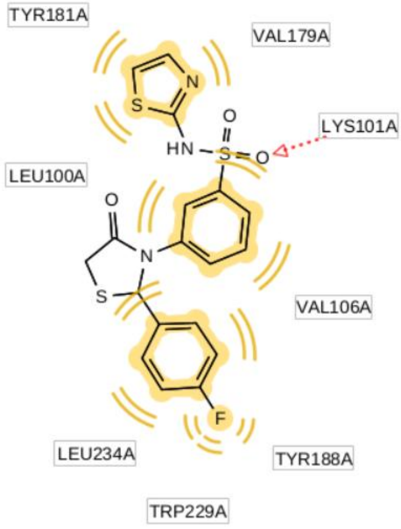

Compound 1

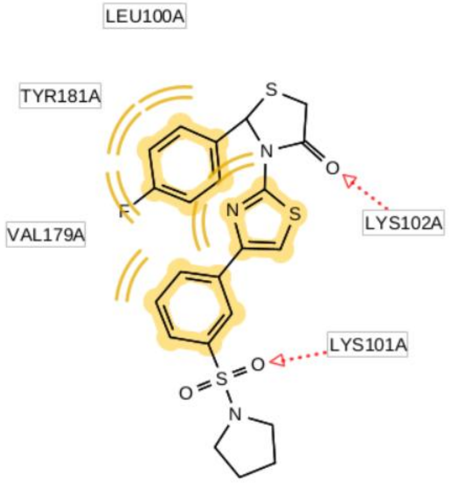

Compound 2

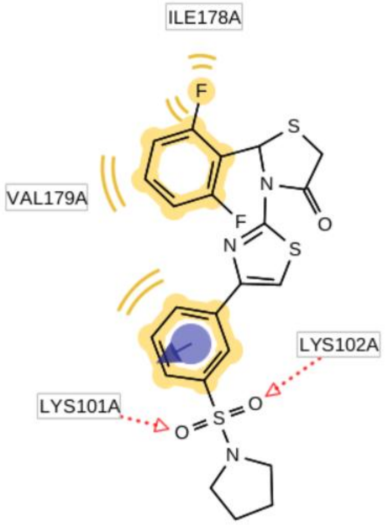

Compound 3

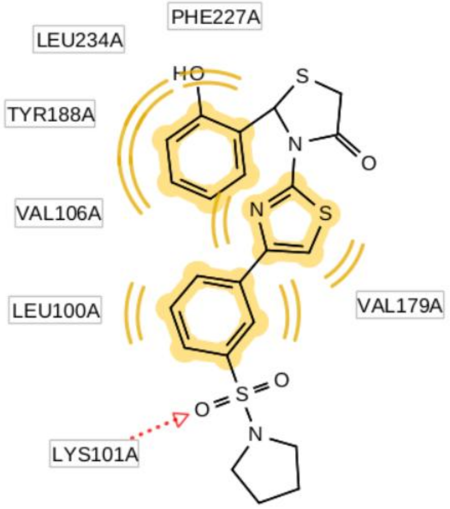

Compound 5

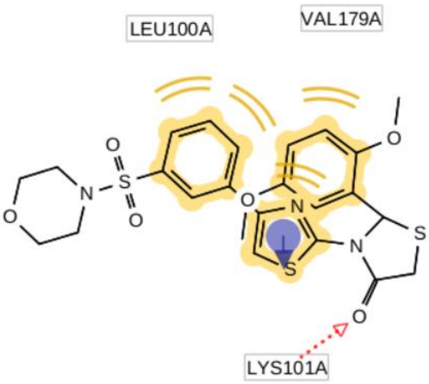

Compound 6

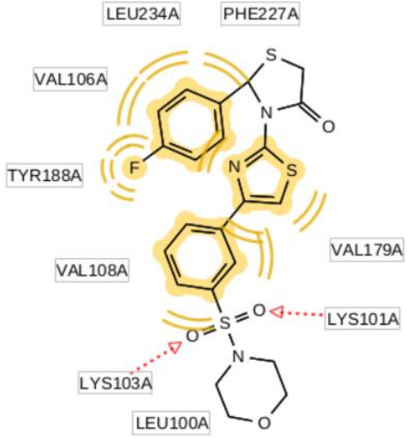

Compound 7

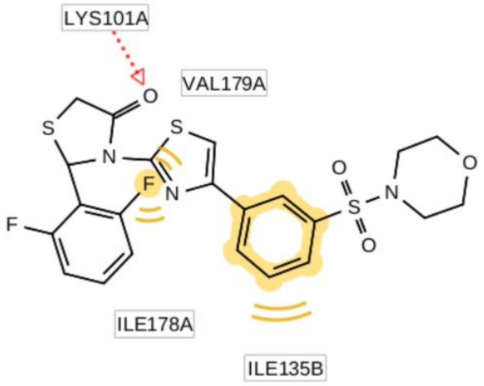

Compound 8

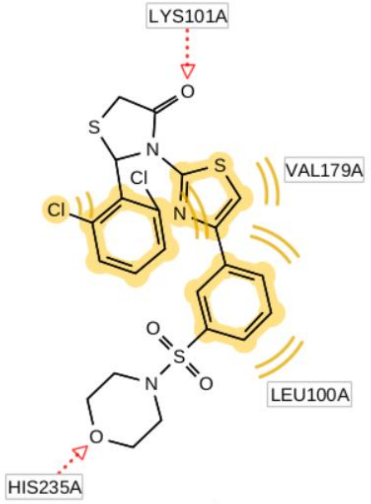

Compound 9

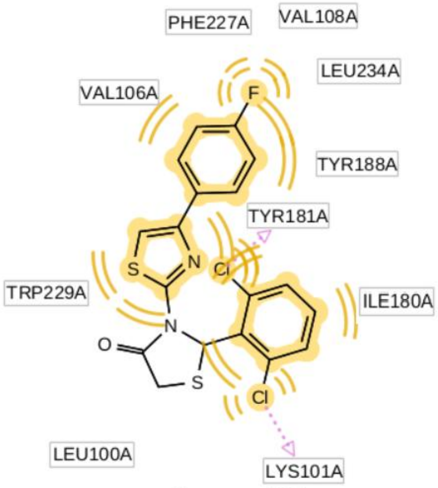

Compound 10

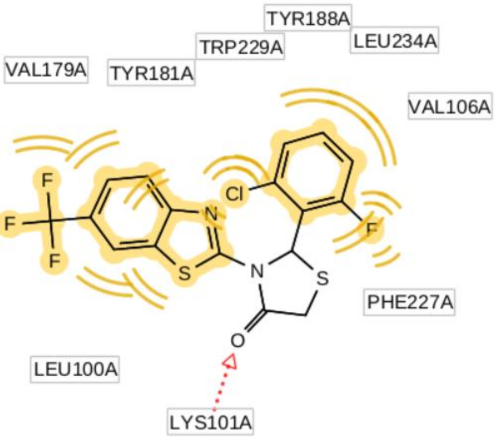

Compound 11

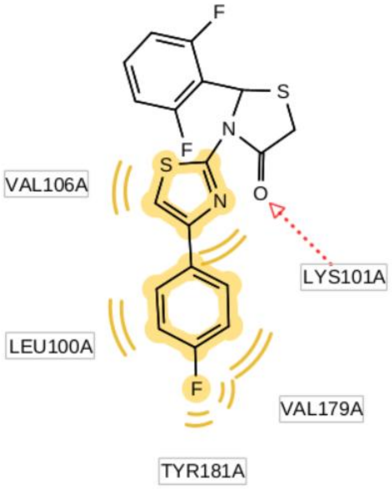

Compound 12

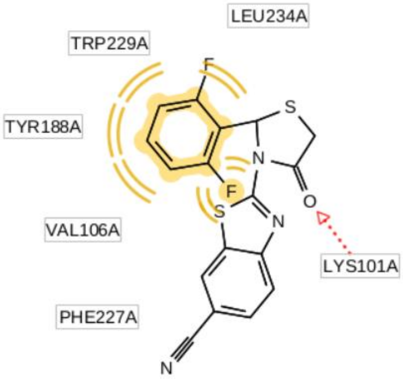

Compound 13

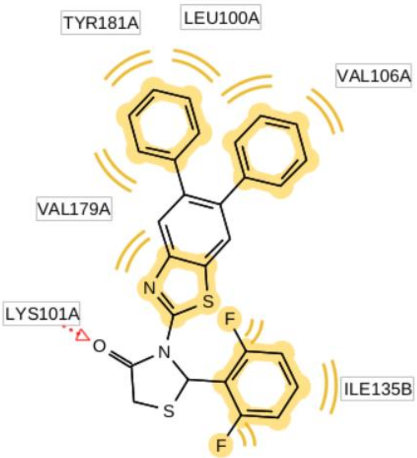

Compound 14

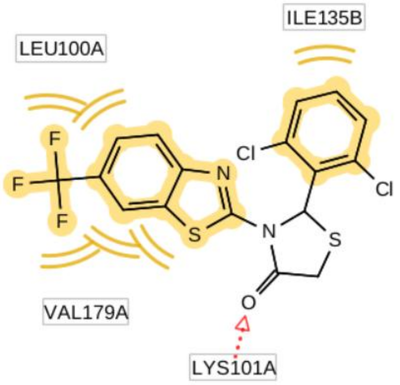

Compound 15

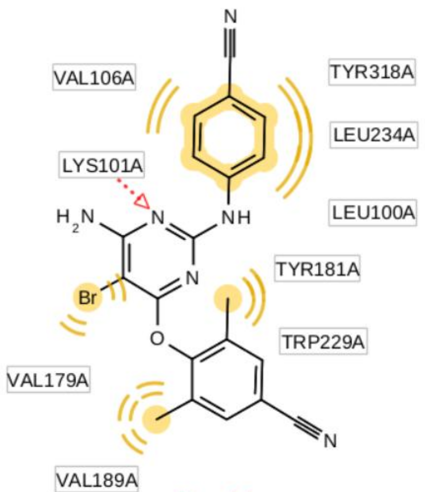

Etravirine

5. **2D interaction diagrams of compounds 1–15 and reference compounds with the RNAase active site**

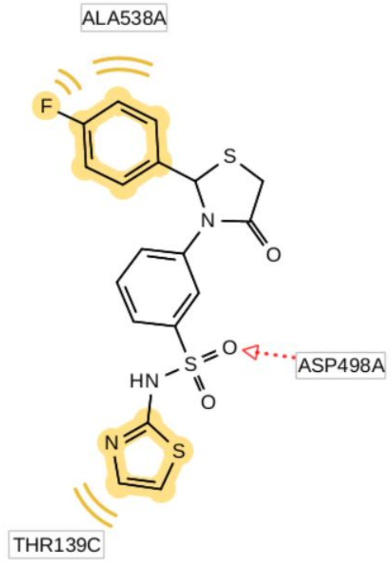

Compound 1

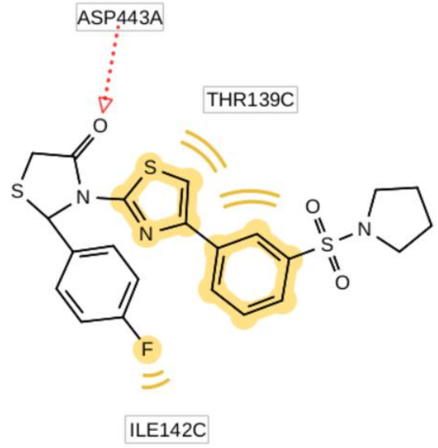

Compound 2

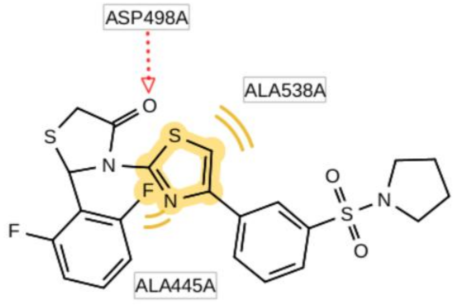

Compound 3

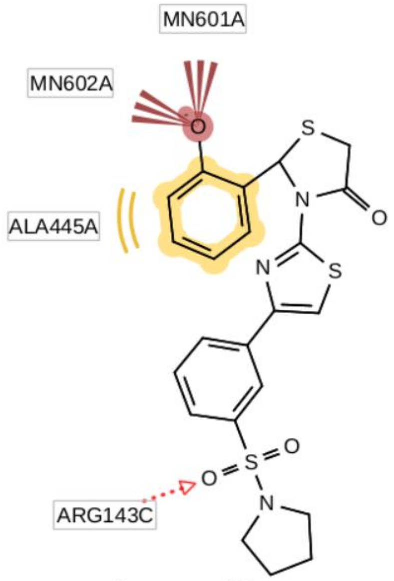

Compound 5

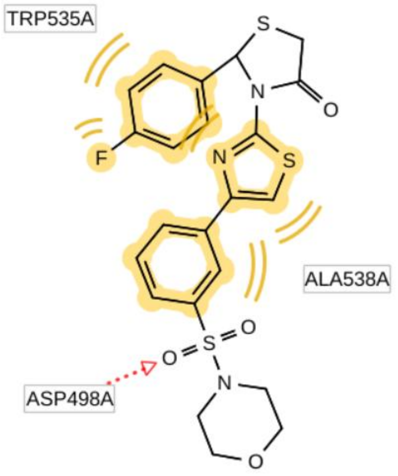

Compound 7

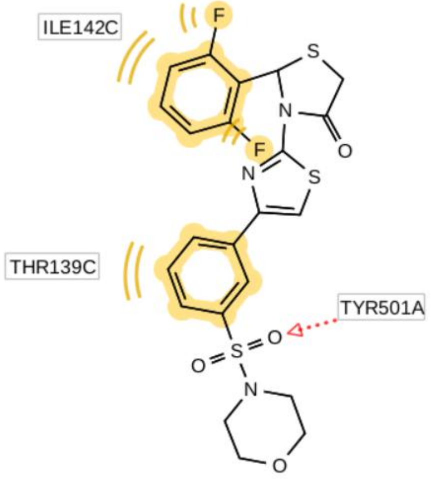

Compound 8

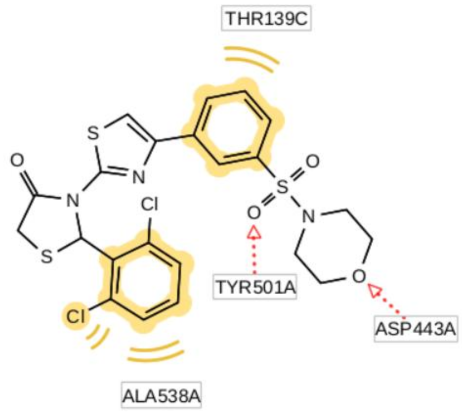

Compound 9

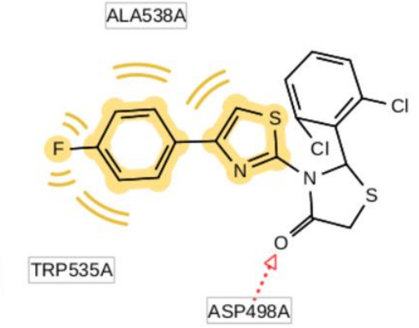

Compound 10

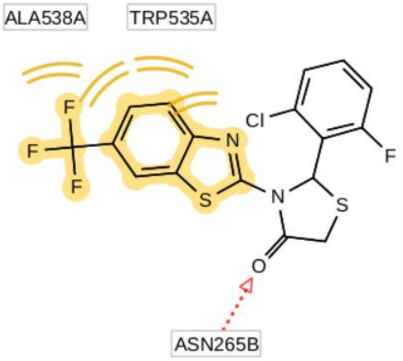

Compound 11

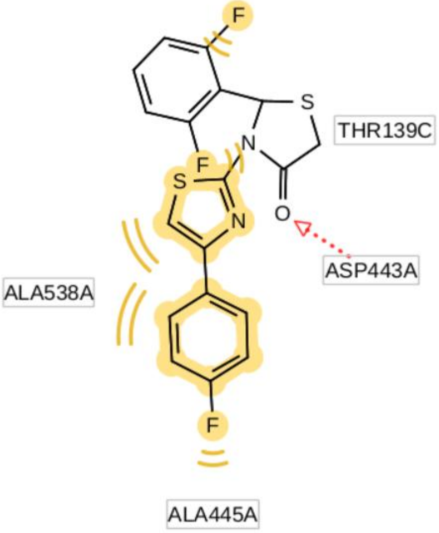

Compound 12

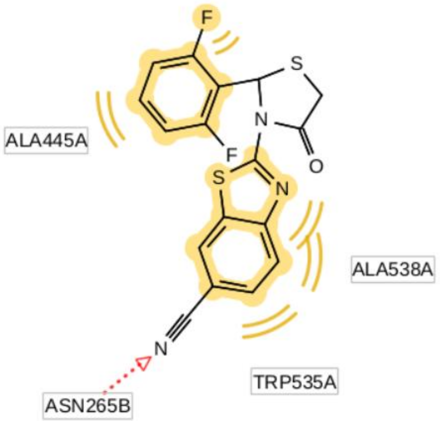

Compound 13

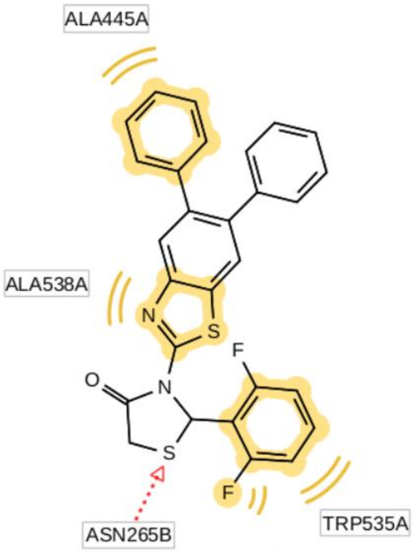

Compound 14

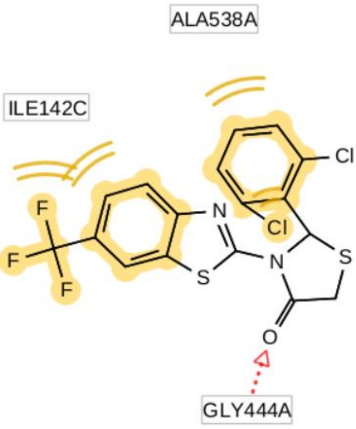

Compound 15

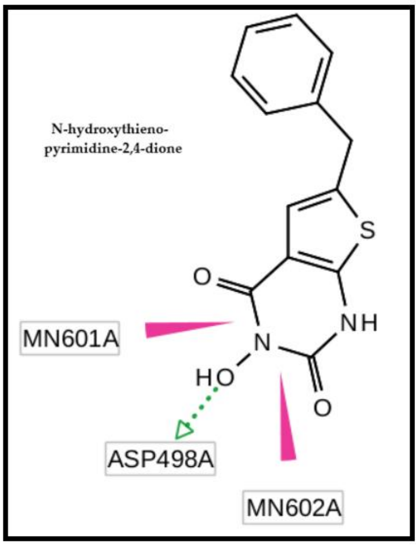

6. ^{1}H NMR and ^{13}C NMR of compunds

Compound 2

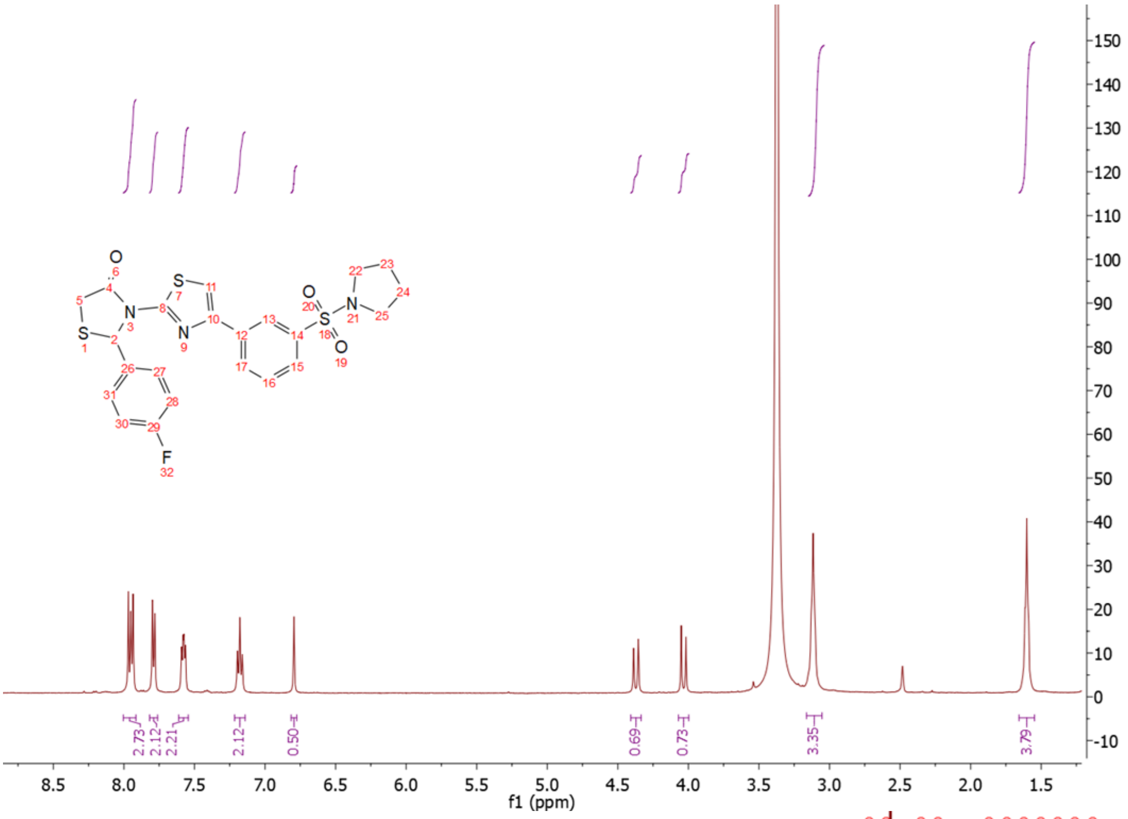

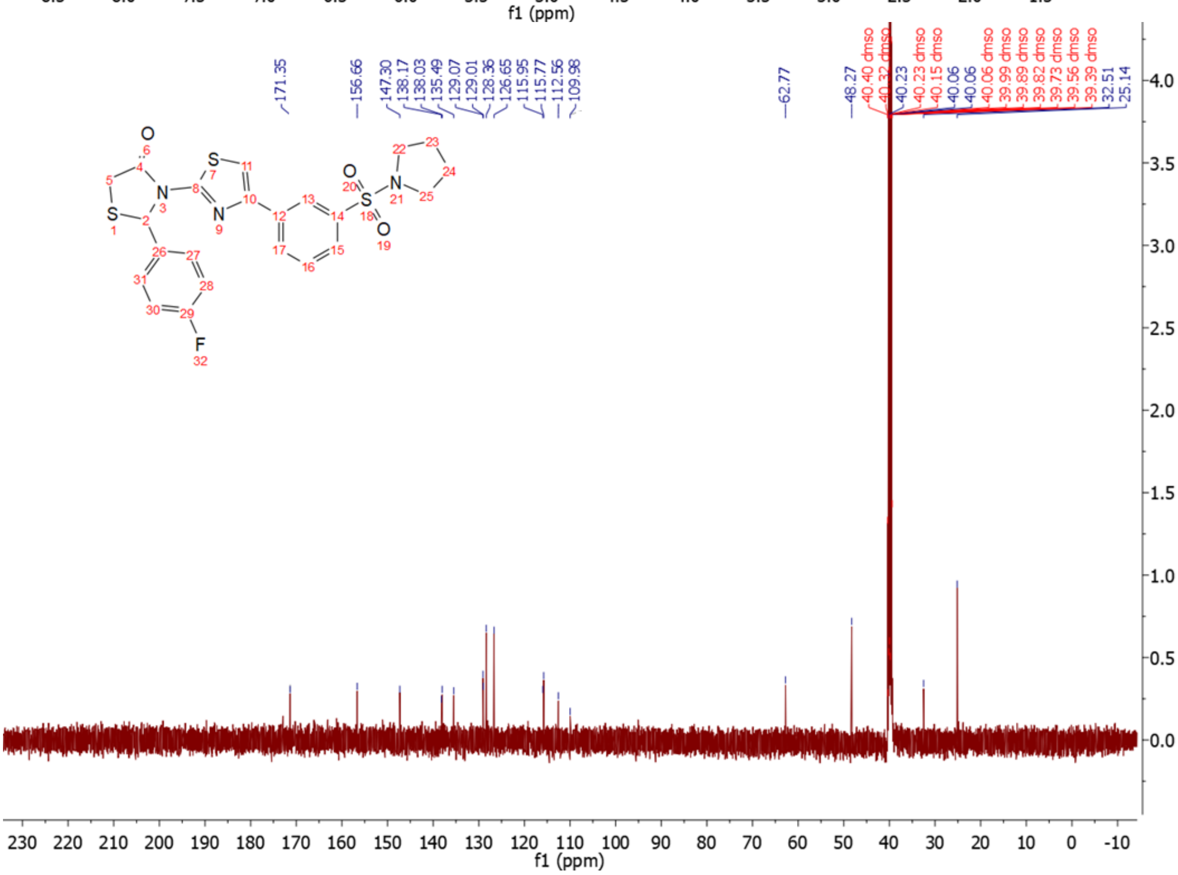

Compound 3

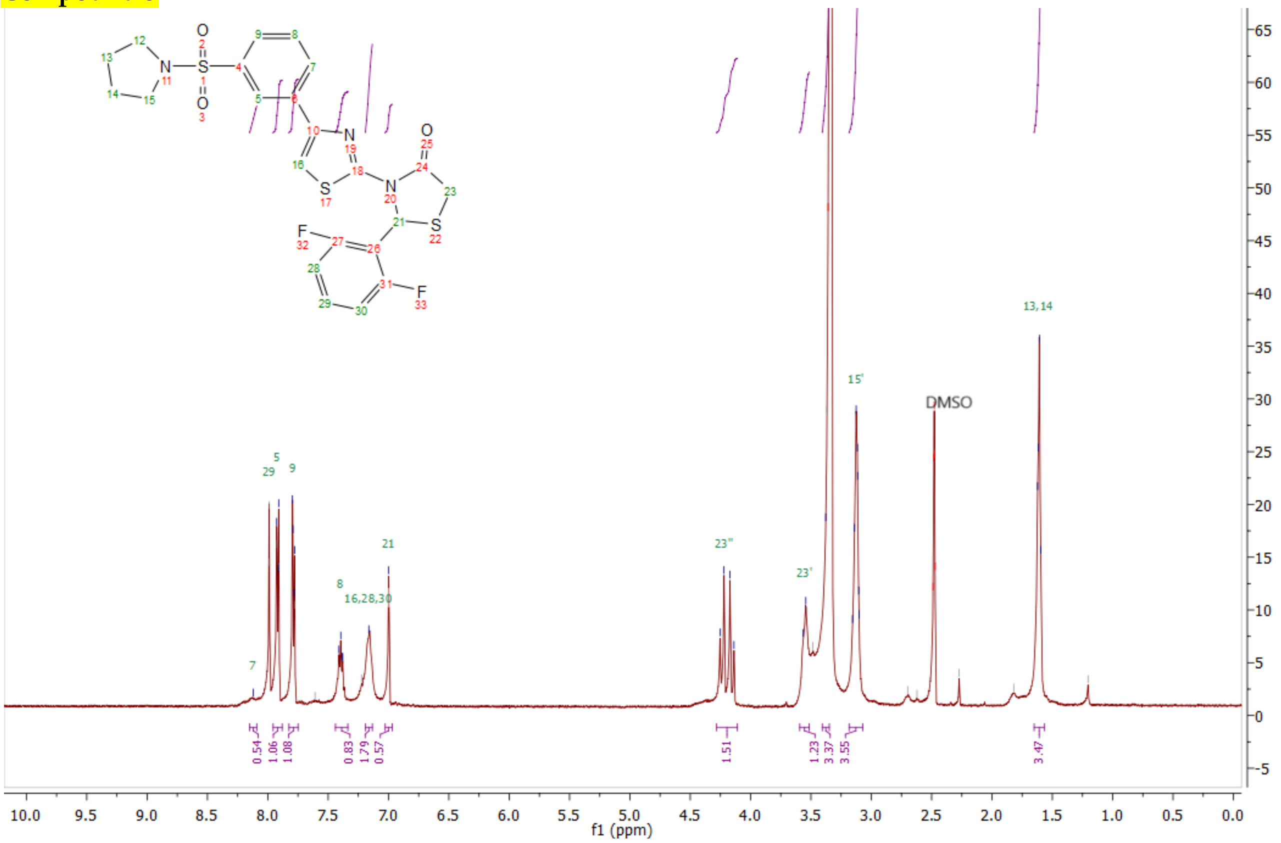

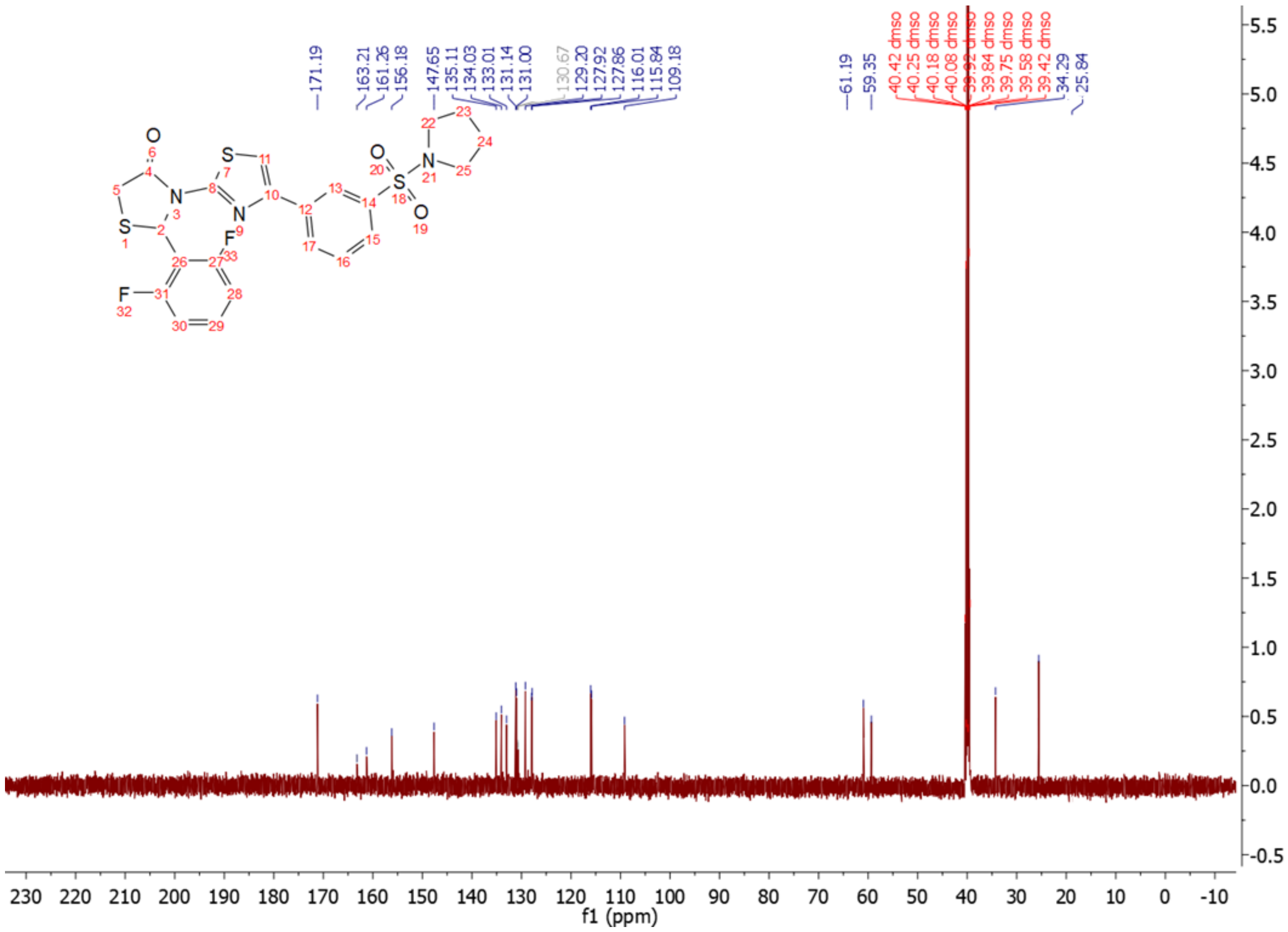

Compound 4

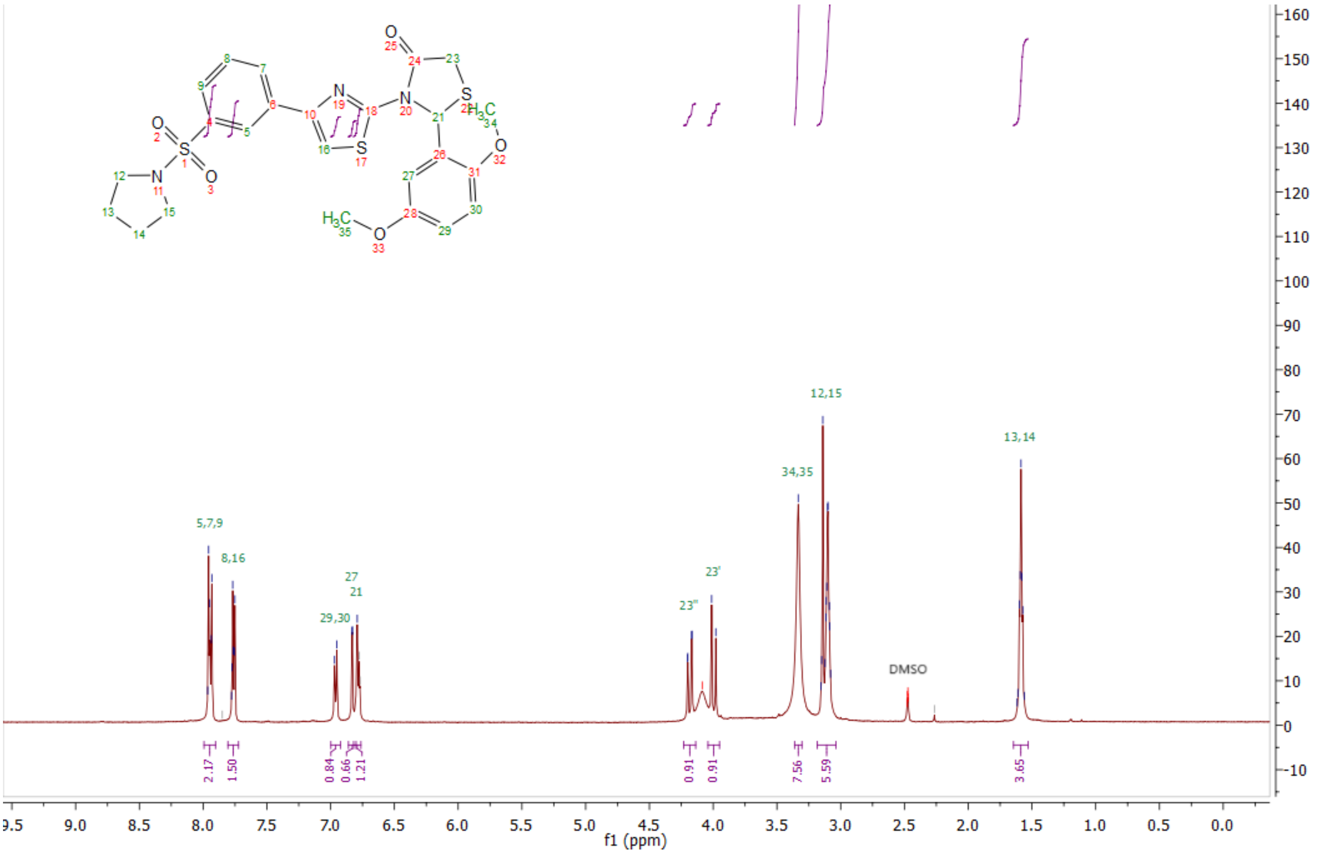
5,7,9
8,16
29,30
27
21
23"
23'
34,35
12,15
DMSO
13,14
2.17
1.50
0.84
0.66
1.21
0.91
0.91
7.56
5.59
3.65
f1 (ppm)

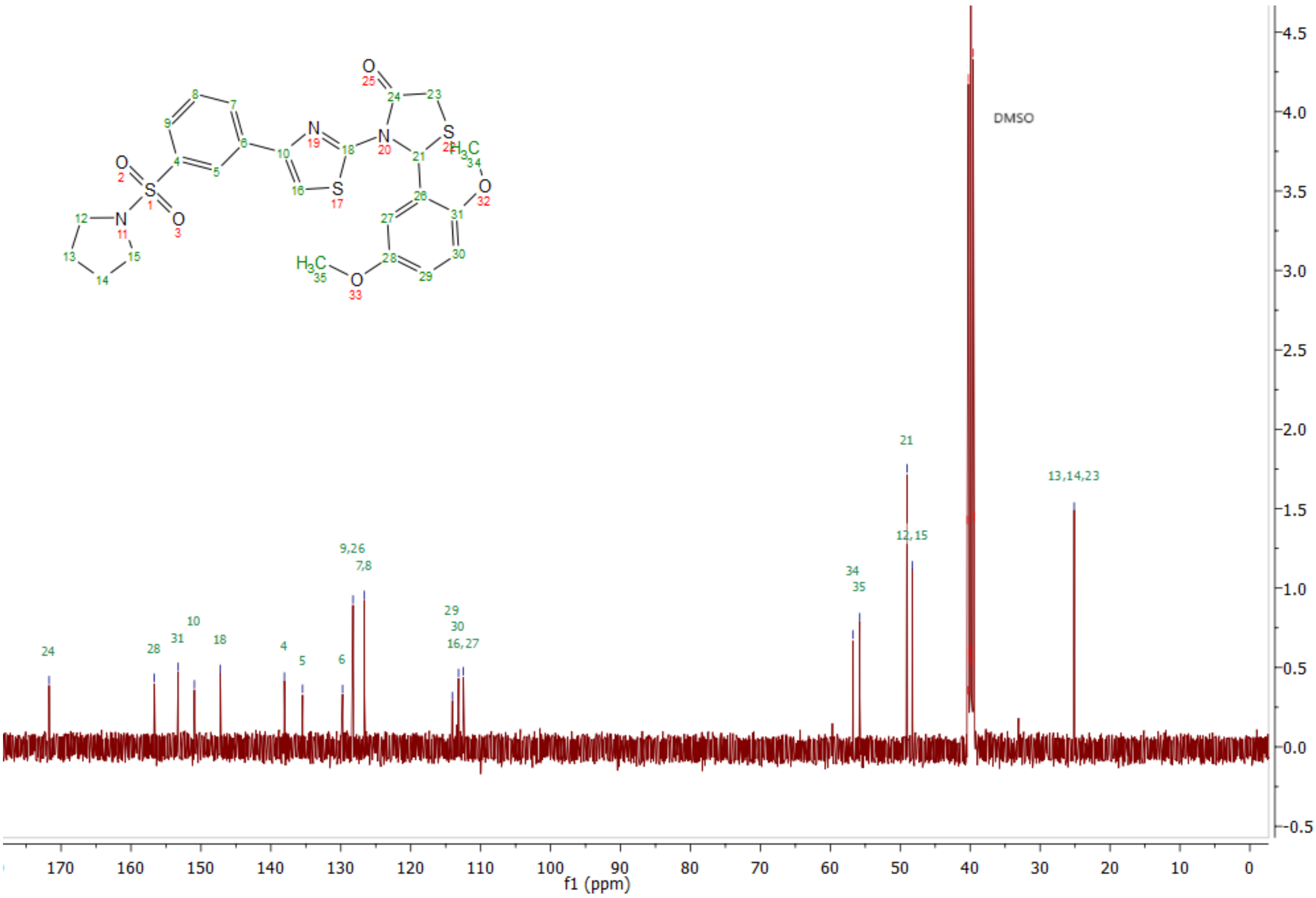
DMSO
21
13,14,23
12,15
34
35
9,26
7,8
29
30
16,27
24
28
31
10
18
4
5
6
f1 (ppm)

Compound 5

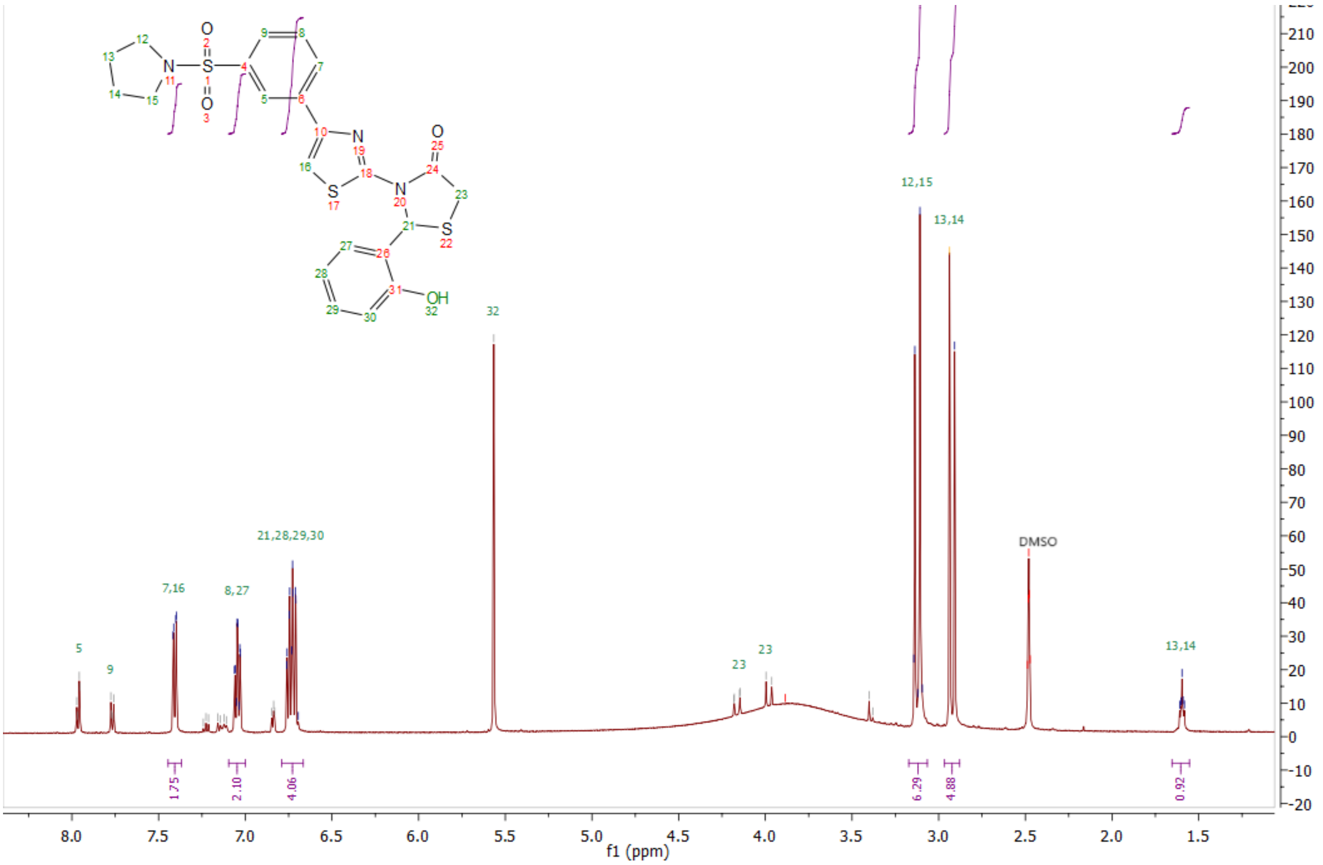
12,15
13,14
32
21,28,29,30
7,16
8,27
5
9
23
DMSO
f1 (ppm)

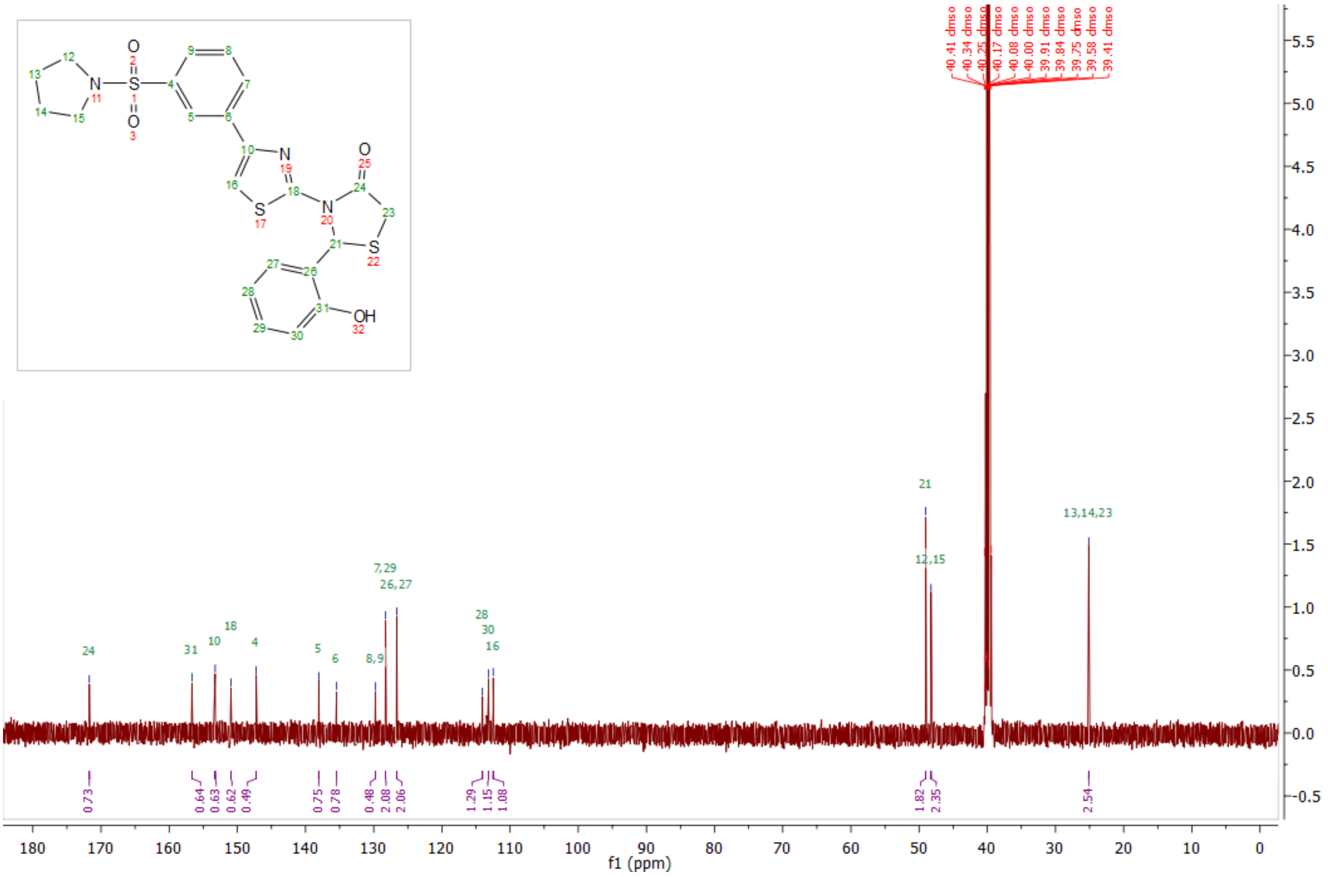
21
12,15
13,14,23
24
31
10
18
4
5
6
8,9
7,29
26,27
28
30
16
f1 (ppm)

Compound 6

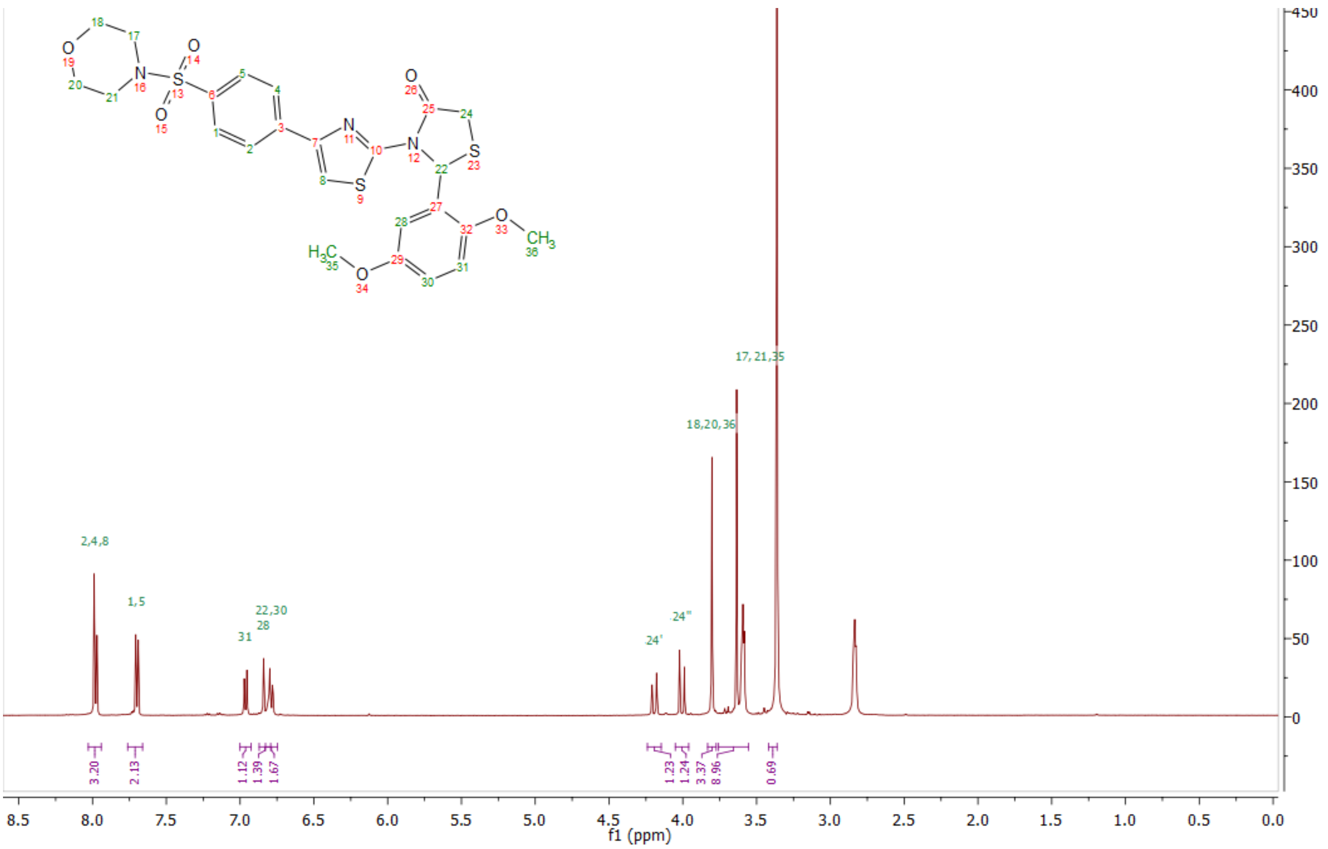

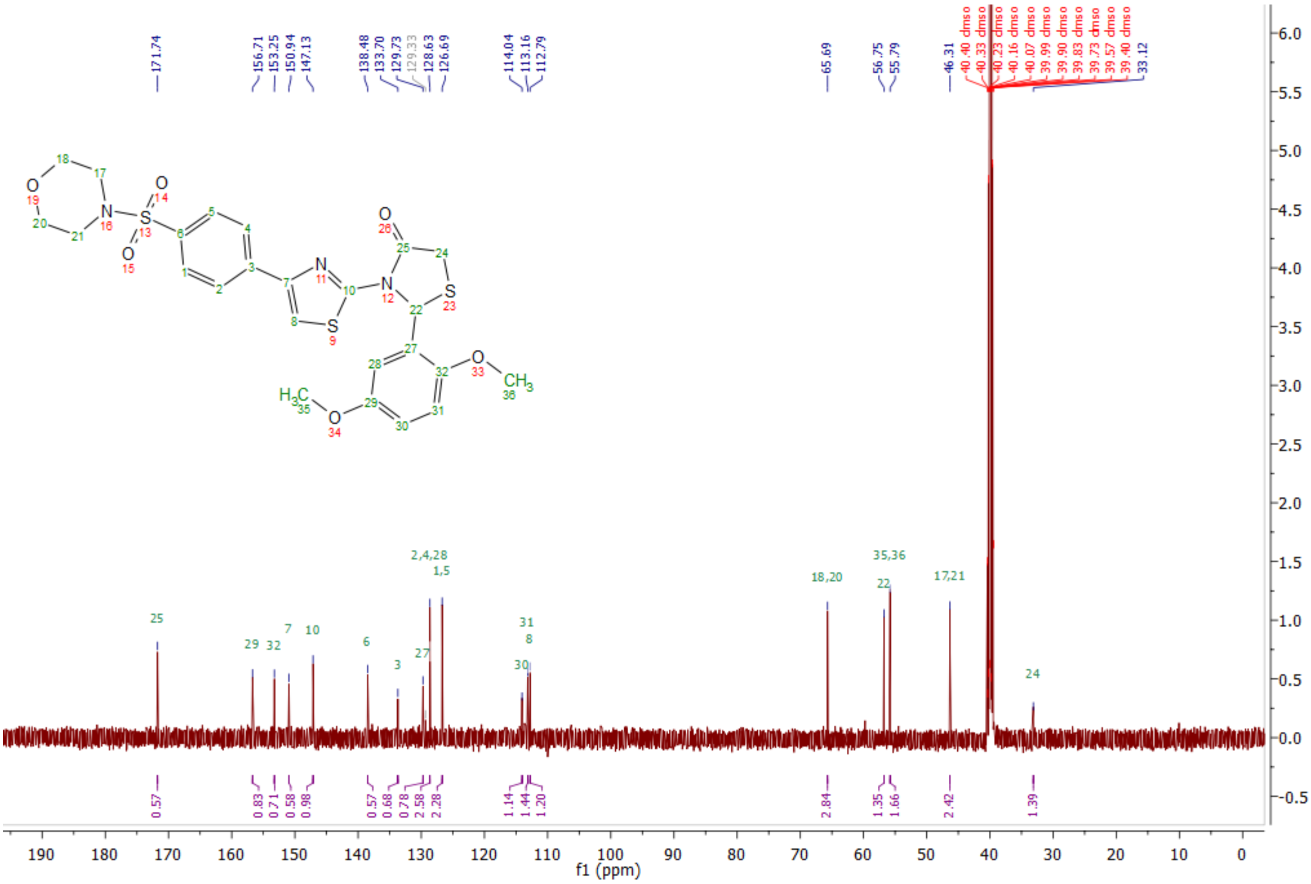

Compound 8

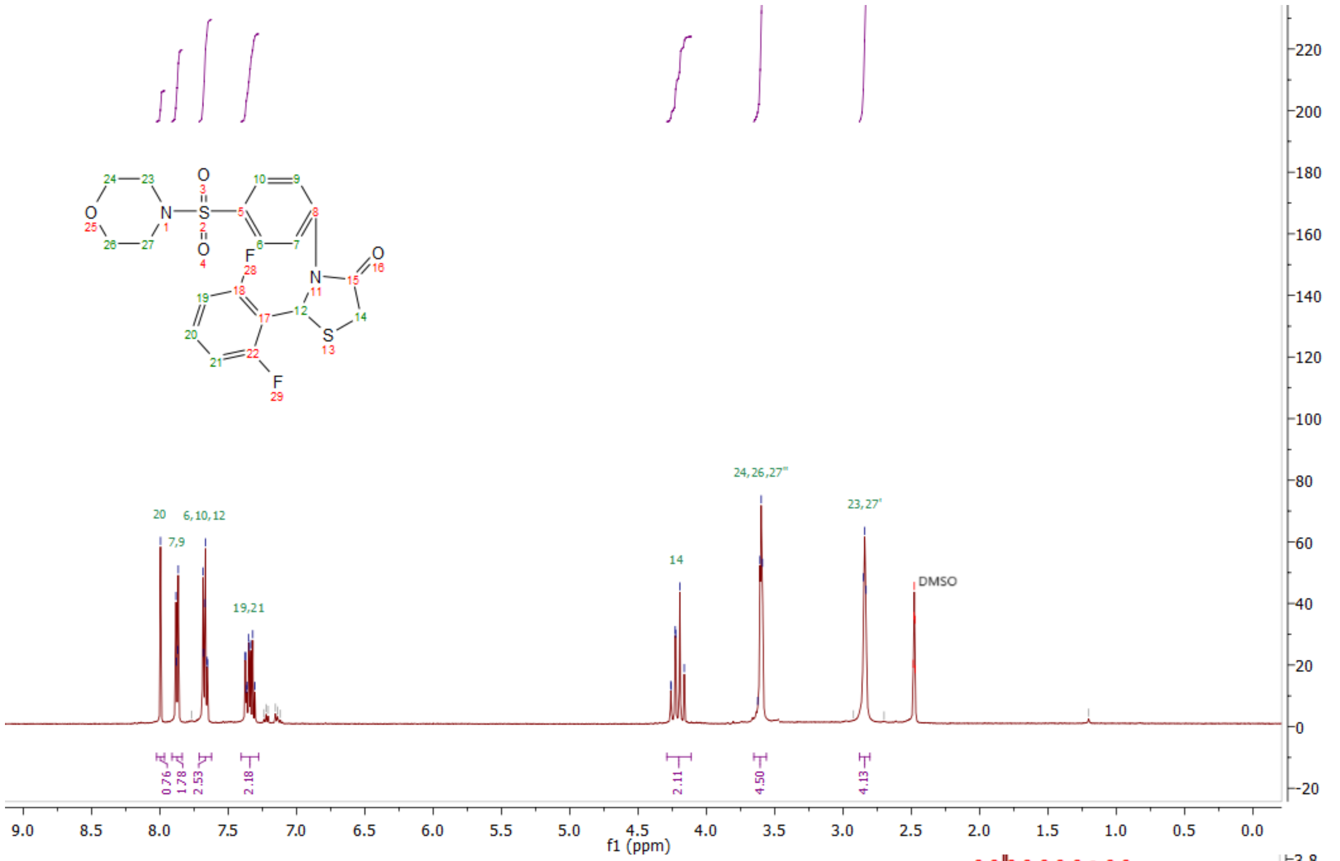
20
7,9
6,10,12
19,21
14
24,26,27''
23,27'
DMSO
0.76
1.78
2.53
2.18
2.11
4.50
4.13
f1 (ppm)

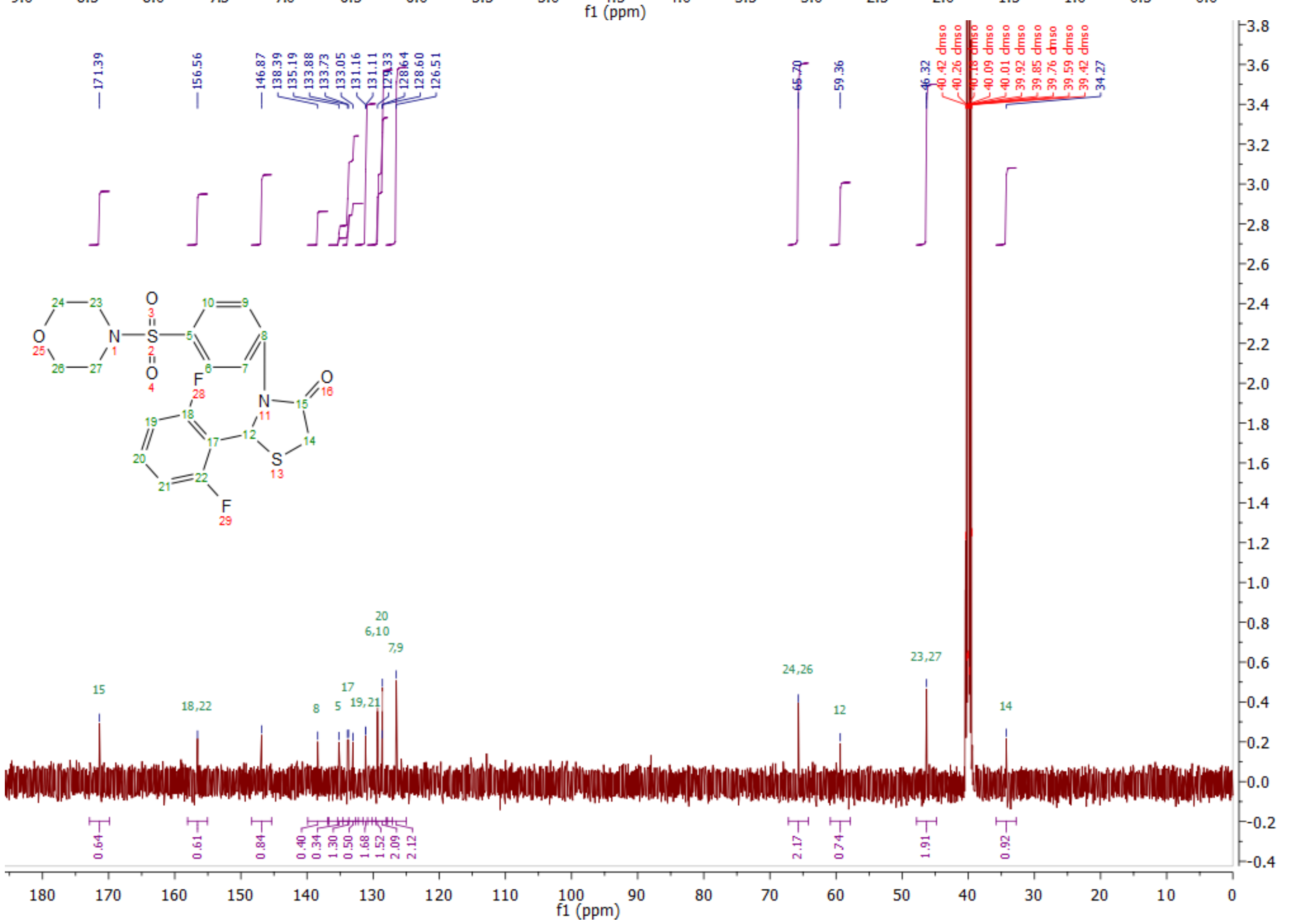
171.39
156.56
146.87
138.39
135.19
133.88
133.73
133.05
131.16
131.11
129.33
128.64
128.60
126.51
65.70
59.36
46.32
34.27
15
18,22
8
5
17
19,21
20
6,10
7,9
24,26
12
23,27
14
0.64
0.61
0.84
2.17
0.74
1.91
0.92
f1 (ppm)

Compound 10

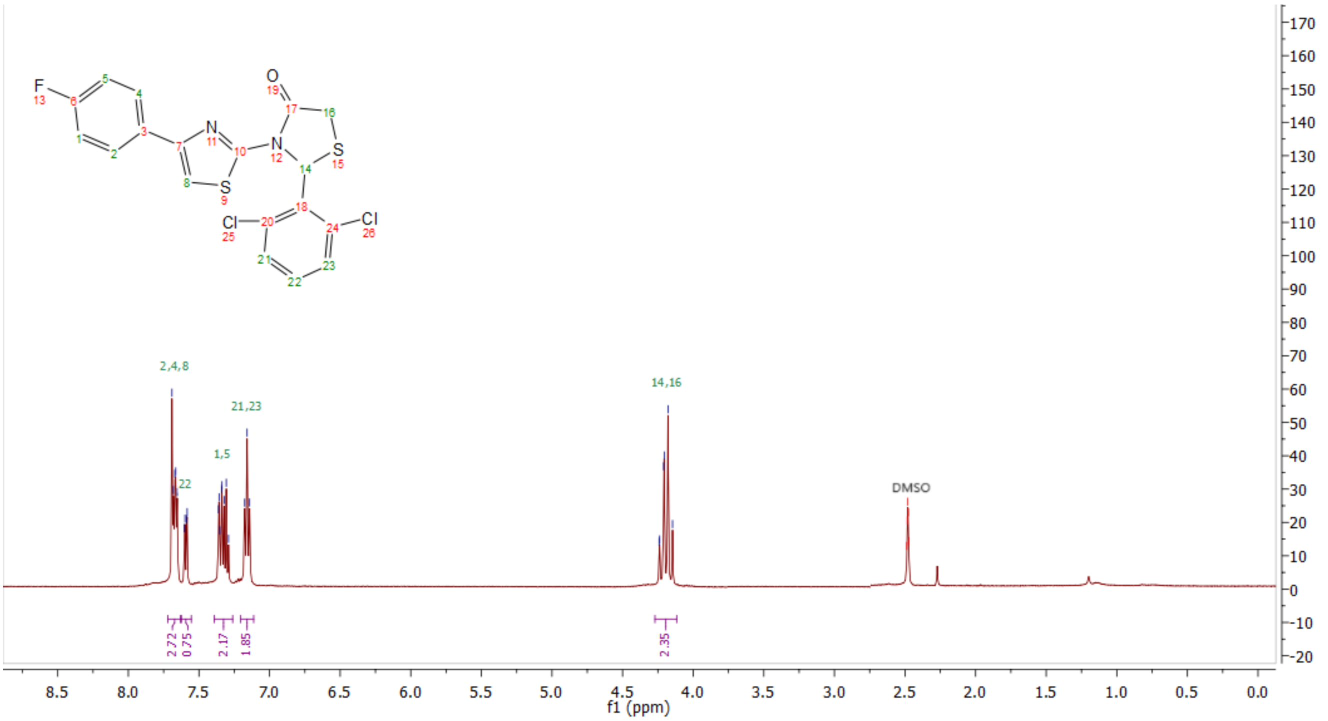

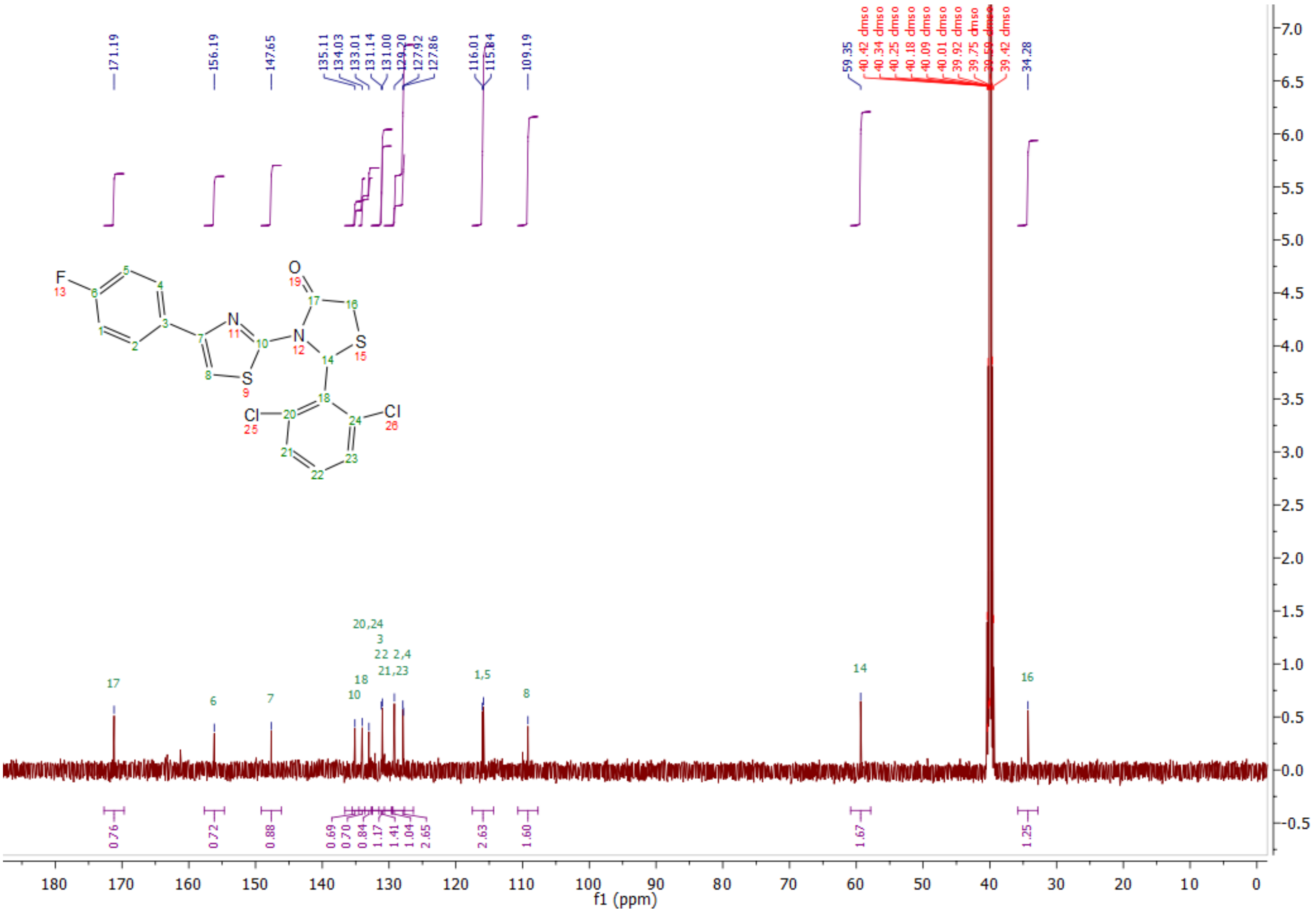

Compound 11

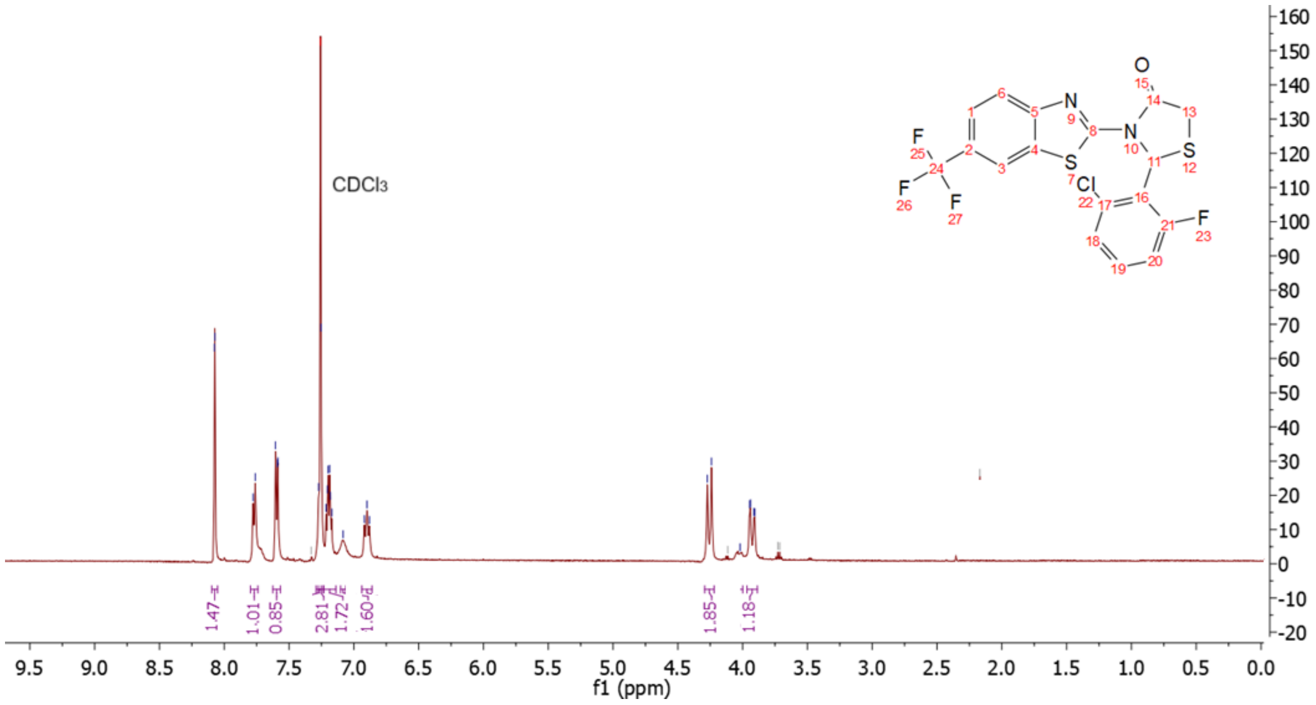

CDCl3
1.47
1.01
0.85
2.81
1.72
1.60
1.85
1.18
f1 (ppm)

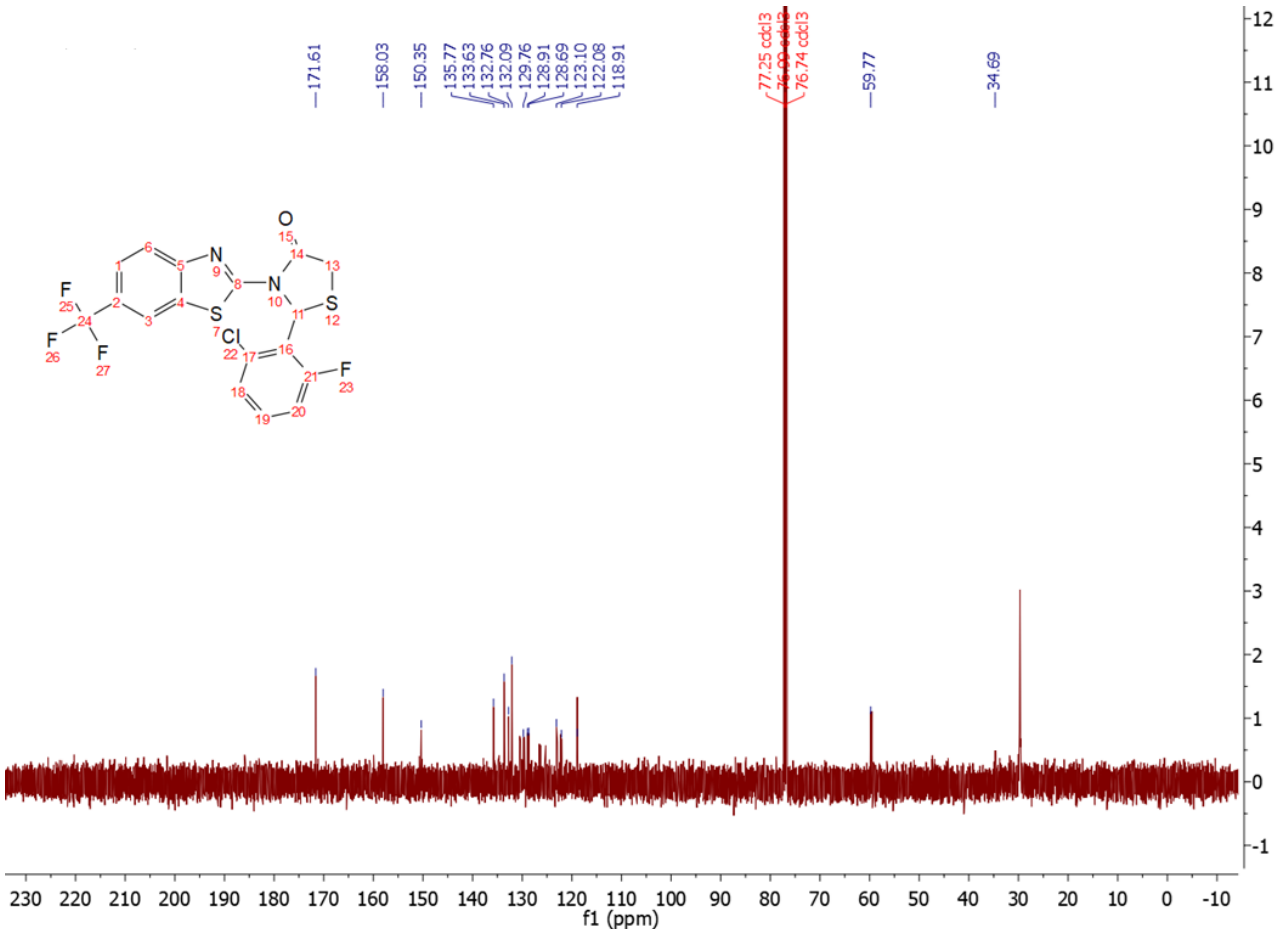

171.61
158.03
150.35
135.77
133.63
132.76
132.09
129.76
128.91
128.69
123.10
122.08
118.91
77.25 cdcl3
76.99 cdcl3
76.74 cdcl3
59.77
34.69
f1 (ppm)

Compound 12

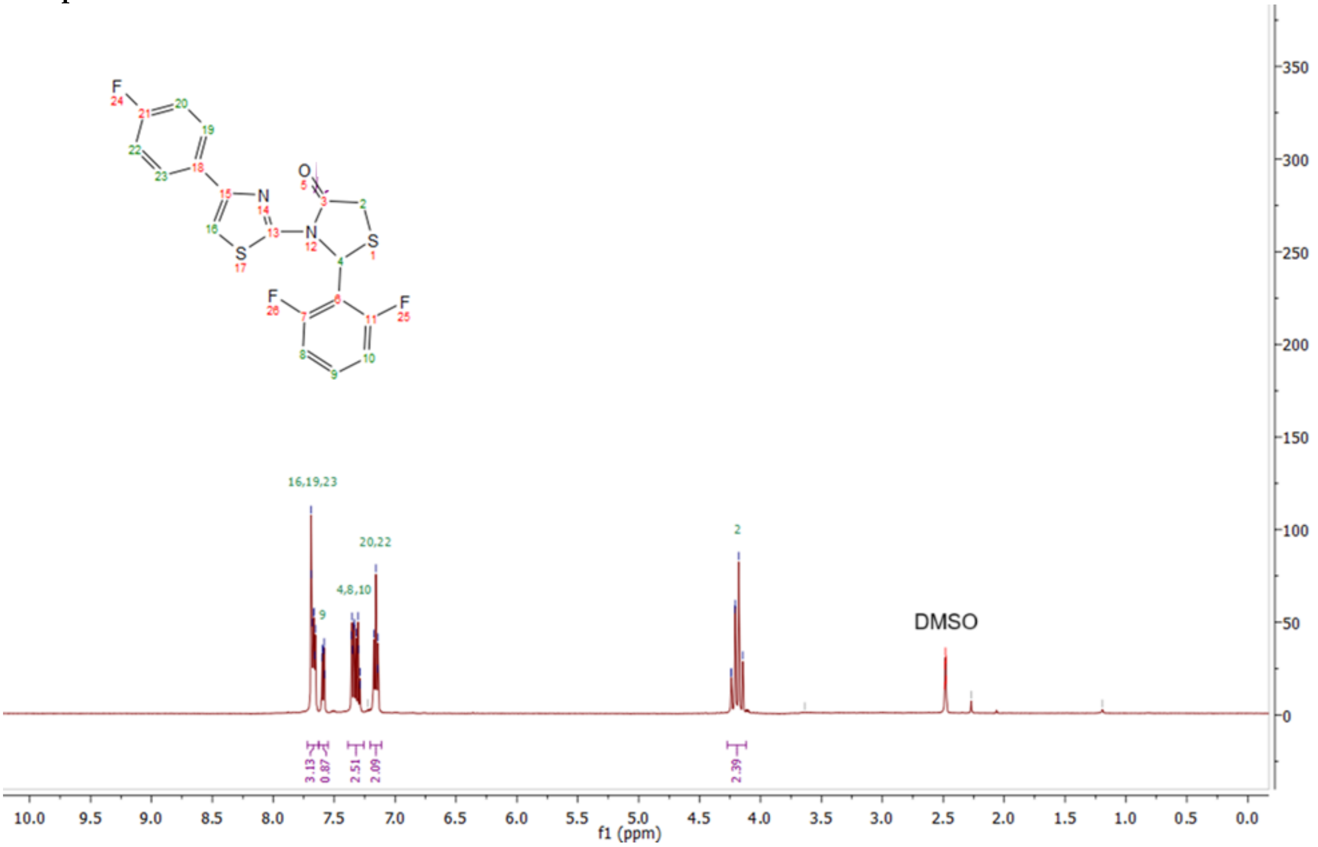
16,19,23
9
4,8,10
20,22
2
DMSO
3.13
0.87
2.51
2.09
2.39
f1 (ppm)

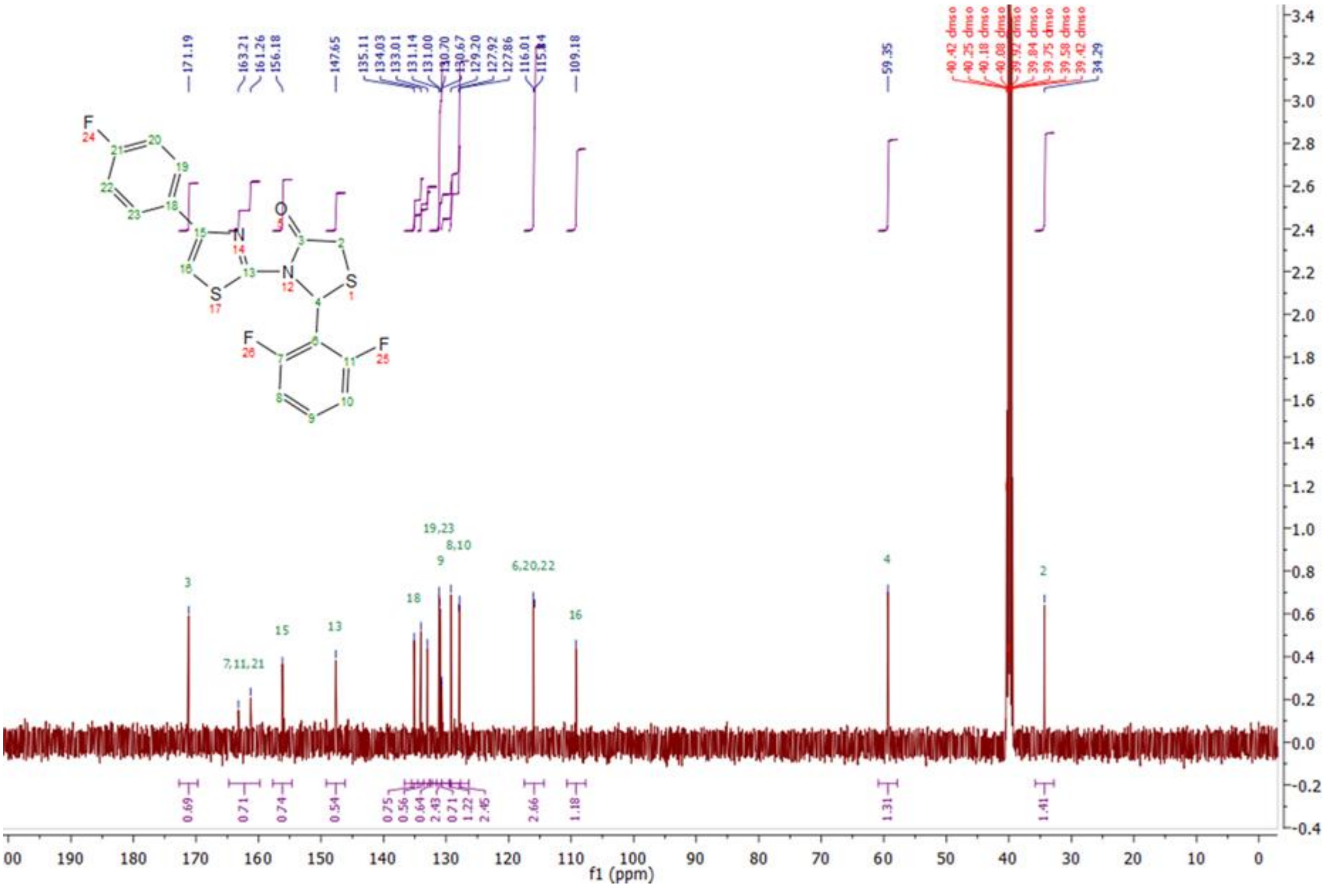
171.19
163.21
161.26
156.18
147.65
135.11
134.03
133.01
131.14
131.00
130.70
130.67
129.20
127.92
127.86
116.01
109.18
59.35
40.42 dmso
40.25 dmso
40.18 dmso
40.08 dmso
39.92 dmso
39.84 dmso
39.75 dmso
39.58 dmso
39.42 dmso
34.29
3
7,11,21
15
13
18
19,23
8,10
9
6,20,22
16
4
2
0.69
0.71
0.74
0.54
0.75
0.56
0.64
2.43
0.71
1.22
2.45
2.66
1.18
1.31
1.41
f1 (ppm)

Compound 14

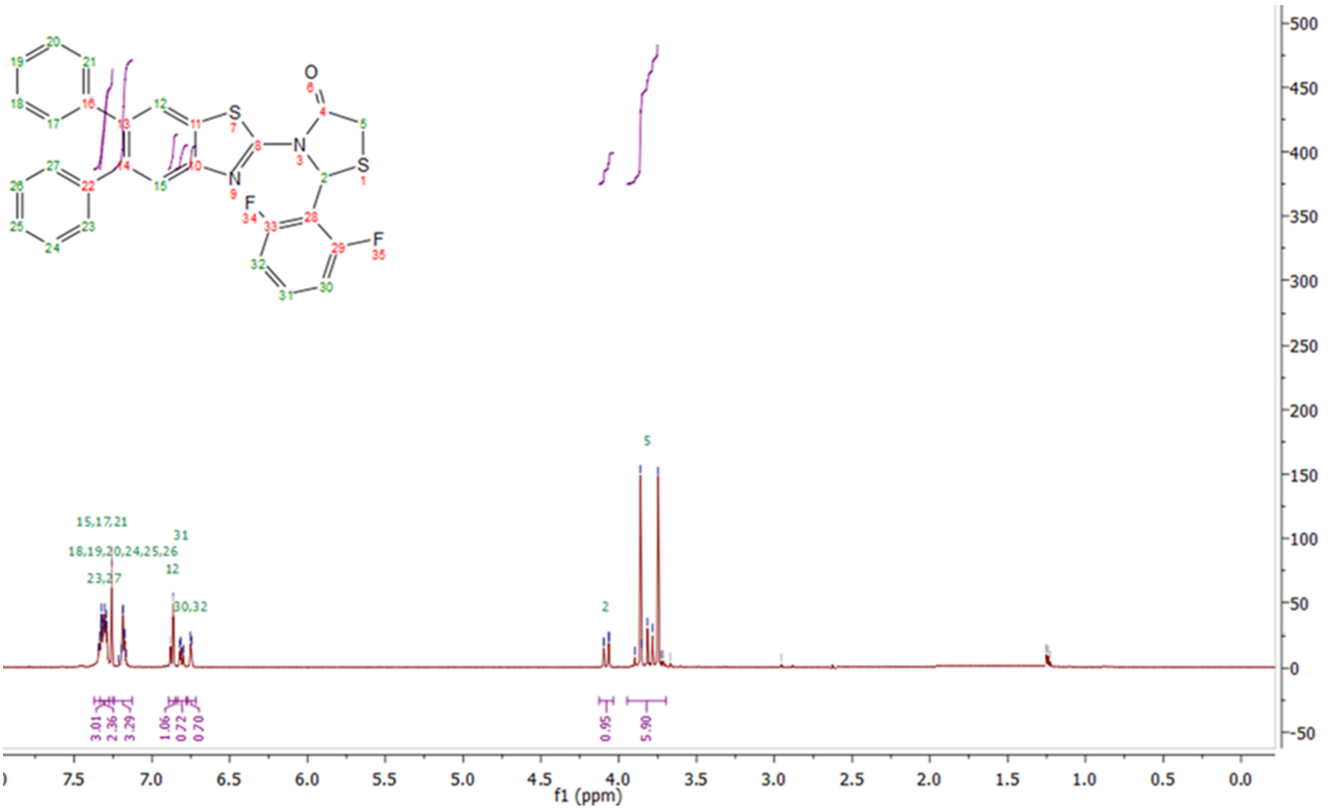
15,17,21
18,19,20,24,25,26
23,27
31
12
30,32
2
5
3.01
2.36
3.29
1.06
0.72
0.70
0.95
5.90
7.5 7.0 6.5 6.0 5.5 5.0 4.5 4.0 3.5 3.0 2.5 2.0 1.5 1.0 0.5 0.0
f1 (ppm)
500 450 400 350 300 250 200 150 100 50 0 -50

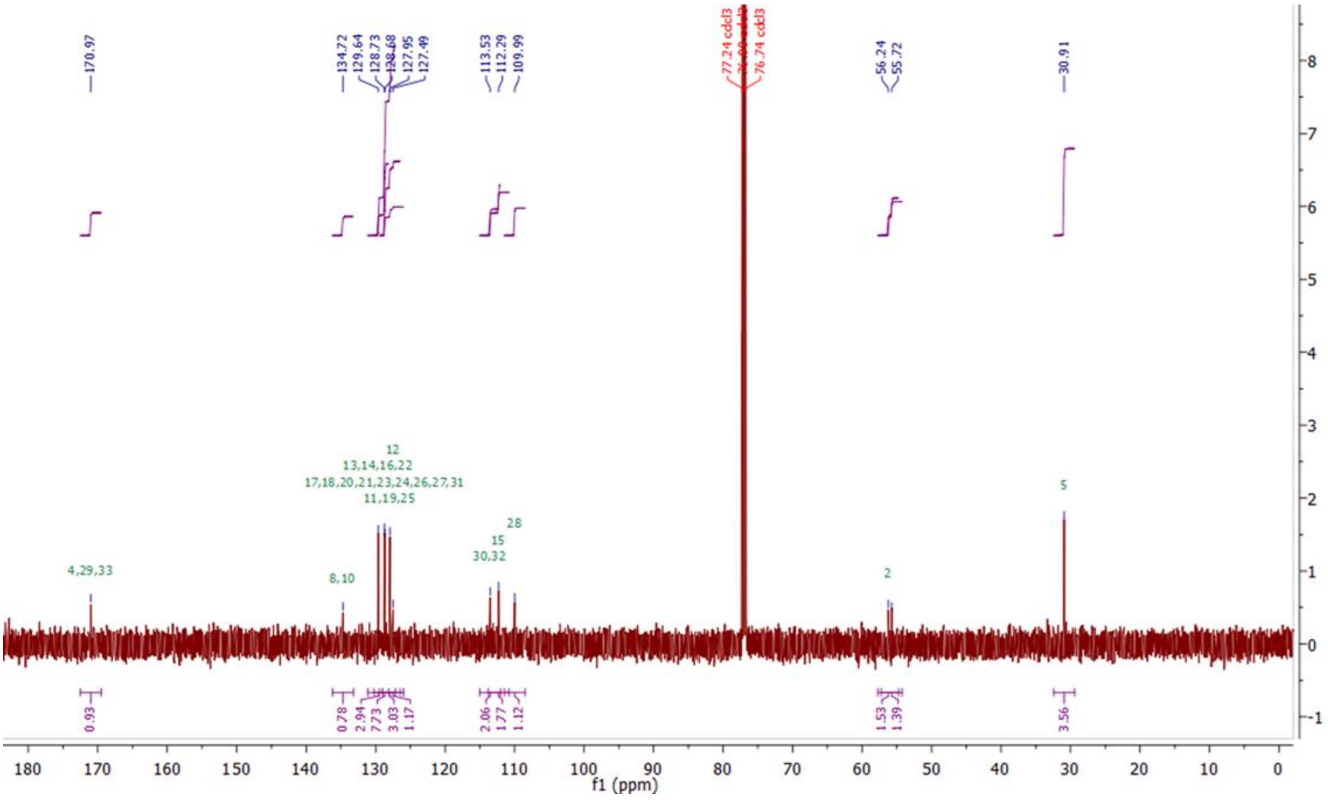
170.97
134.72
129.64
128.73
128.68
127.95
127.49
113.53
112.29
109.99
77.24 cdcl3
76.74 cdcl3
56.24
55.72
30.91
4,29,33
8,10
12
13,14,16,22
17,18,20,21,23,24,26,27,31
11,19,25
28
15
30,32
2
5
0.93
0.78
2.94
7.73
3.03
1.17
2.06
1.77
1.12
1.53
1.39
3.56
180 170 160 150 140 130 120 110 100 90 80 70 60 50 40 30 20 10 0
f1 (ppm)

Compound 15

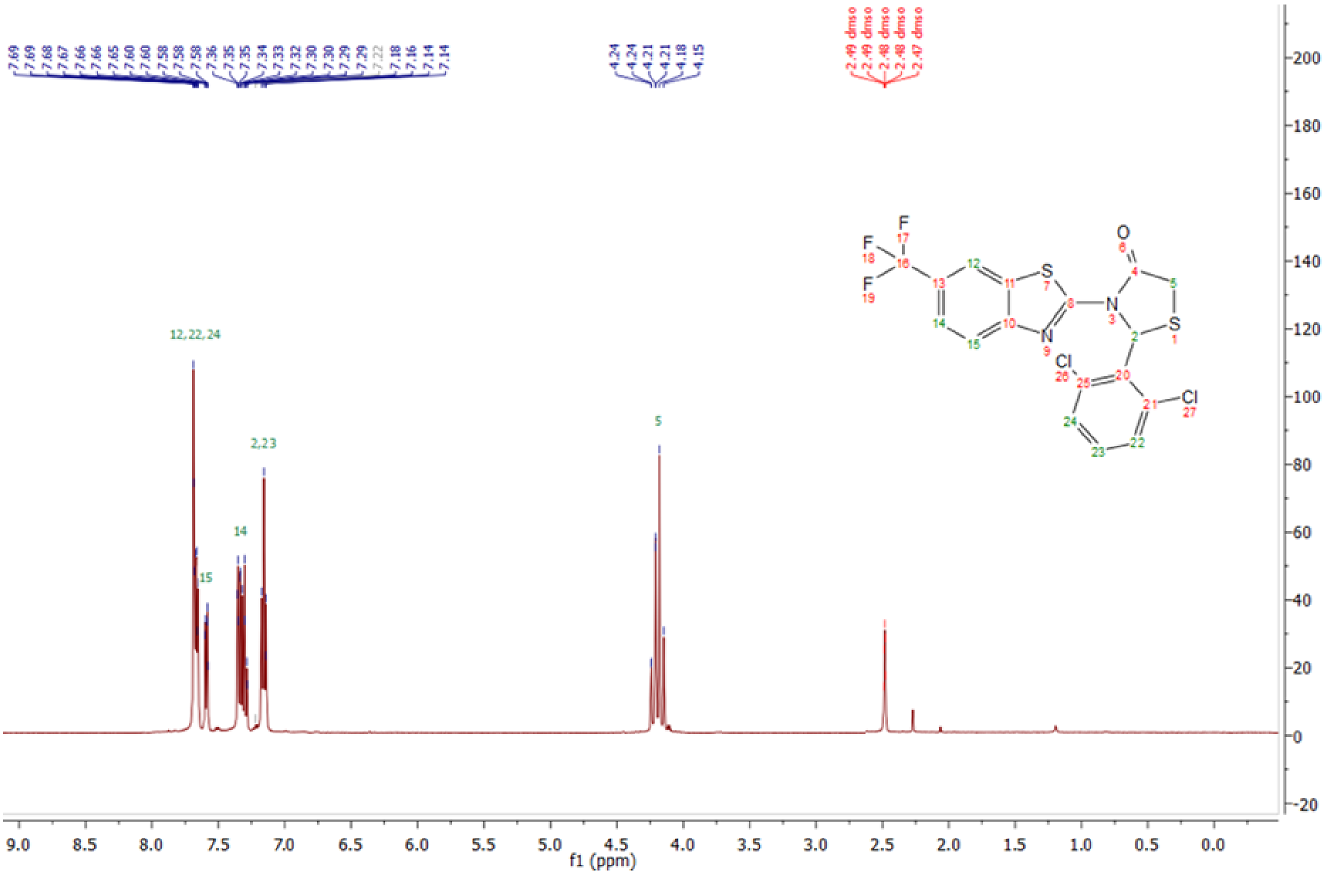

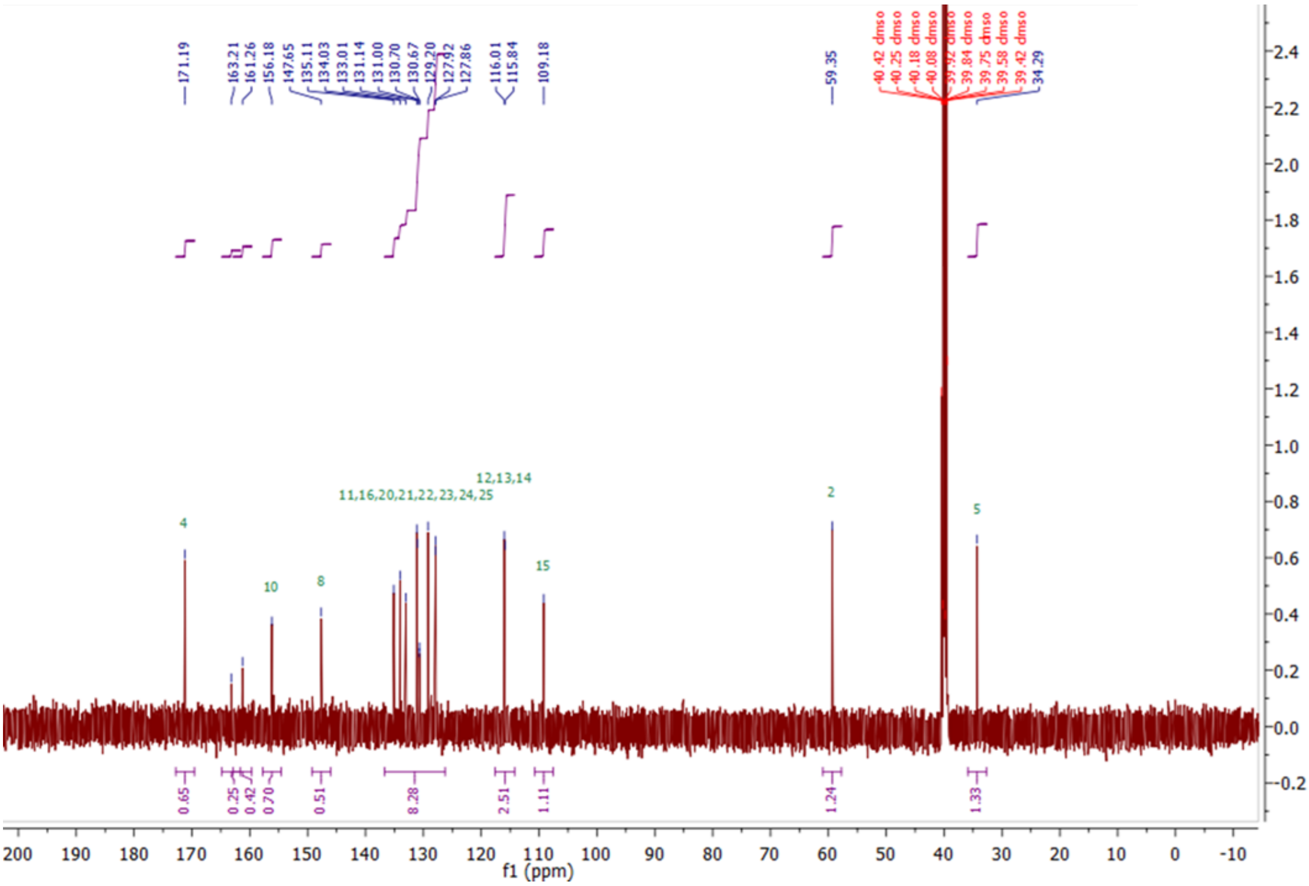

8. MRC-5 cell line

The previously established human normal MRC-5 (human fetal lung fibroblasts; RRID:CVCL_0440) approved for vaccine production was obtained, stored and used in a routine manner (1,2) in the Laboratory of Pharmacology, School of Pharmacy, Aristotle University of Thessaloniki, Greece (Laboratory of Ioannis S. Vizirianakis, Associate Professor in Molecular Pharmacology and Pharmacogenomics) (<25 passages was applied).

References

1. Tseligka ED, Rova A, Amanatiadou EP, Calabrese G, Tsibouklis J, Fatouros DG and Vizirianakis IS. (2016). Pharmacological development of target-specific delocalized lipophilic cation-functionalized carboranes for cancer therapy. Pharm. Res. 33: 1945-1958. doi: 10.1007/s11095-016-1930-4.

2. Akrivou MG, Demertzidou VP, Theodoroula NF, Chatzopoulou FM, Kyritsis KA, Grigoriadis N, Zografos AL, Vizirianakis IS. (2018). Uncovering the pharmacological response of novel sesquiterpene derivatives that differentially alter gene expression and modulate the cell cycle in cancer cells. Int. J. Oncol. 53(5):2167-2179. doi: 10.3892/ijo.2018.4550.

Cell line MRC-5 information

ID/Proper Citation: (RRID:CVCL_0440)

URL: https://web.expasy.org/cellosaurus/CVCL_0440
